# Gram Negative Biofilms: Structural and Functional Responses to Destruction by Antibiotic-Loaded Mixed Polymeric Micelles

**DOI:** 10.3390/microorganisms12122670

**Published:** 2024-12-23

**Authors:** Tsvetozara Damyanova, Rumena Stancheva, Milena N. Leseva, Petya A. Dimitrova, Tsvetelina Paunova-Krasteva, Dayana Borisova, Katya Kamenova, Petar D. Petrov, Ralitsa Veleva, Ivelina Zhivkova, Tanya Topouzova-Hristova, Emi Haladjova, Stoyanka Stoitsova

**Affiliations:** 1 Department of Microbiology, Stephan Angeloff Institute of Microbiology, Bulgarian Academy of Sciences, Akad. G. Bonchev Street, bl. 26, 1113 Sofia, Bulgaria; tsvetozaradamianova@gmail.com (T.D.); pauny@abv.bg (T.P.-K.); daqanara@abv.bg (D.B.); stoitsova_microbiobas@yahoo.com (S.S.); 2 Institute of Polymers, Bulgarian Academy of Sciences, Akad. G. Bonchev Street, bl. 103-A, 1113 Sofia, Bulgaria; rstancheva@polymer.bas.bg (R.S.); kkamenova@polymer.bas.bg (K.K.); ppetrov@polymer.bas.bg (P.D.P.); 3 Department of Immunology, Stephan Angeloff Institute of Microbiology, Bulgarian Academy of Sciences, Akad. G. Bonchev Street, bl. 26, 1113 Sofia, Bulgariapetya_dimitrova@web.de (P.A.D.); 4 Faculty of Biology, Sofia University “St. Kliment Ohridski”, 8 Dragan Tsankov Blvd., 1164 Sofia, Bulgaria; ralitsa_veleva@biofac.uni-sofia.bg; 5 National Reference Laboratory “Control and Monitoring of Antimicrobial Resistance”, Department of Clinical Microbiology, National Center of Infectious and Parasitic Disease, Yanko Sakuzov Blvd. 26, 1504 Sofia, Bulgaria; ivelinazh@abv.bg

**Keywords:** biofilm destruction, mixed polymer micelles, cationic polymers, drug delivery, biocompatibility, nanotubules, extracellular vesicles, ex vivo skin model

## Abstract

Biofilms are a well-known multifactorial virulence factor with a pivotal role in chronic bacterial infections. Their pathogenicity is determined by the combination of strain-specific mechanisms of virulence and the biofilm extracellular matrix (ECM) protecting the bacteria from the host immune defense and the action of antibacterials. The successful antibiofilm agents should combine antibacterial activity and good biocompatibility with the capacity to penetrate through the ECM. The objective of the study is the elaboration of biofilm-ECM-destructive drug delivery systems: mixed polymeric micelles (MPMs) based on a cationic poly(2-(dimethylamino)ethyl methacrylate)-b-poly(ε-caprolactone)-b-poly(2-(dimethylamino)ethyl methacrylate) (PDMAEMA_35_-b-PCL_70_-b-PDMAEMA_35_) and a non-ionic poly(ethylene oxide)-b-poly(propylene oxide)-b-poly(ethylene oxide) (PEO_100_-b-PPO_65_-b-PEO_100_) triblock copolymers, loaded with ciprofloxacin or azithromycin. The MPMs were applied on 24 h pre-formed biofilms of *Escherichia coli* and *Pseudomonas aeruginosa* (laboratory strains and clinical isolates). The results showed that the MPMs were able to destruct the biofilms, and the viability experiments supported drug delivery. The biofilm response to the MPMs loaded with the two antibiotics revealed two distinct patterns of action. These were registered on the level of both bacterial cell-structural alterations (demonstrated by scanning electron microscopy) and the interaction with host tissues (ex vivo biofilm infection model on skin samples with tests on nitric oxide and interleukin (IL)-17A production).

## 1. Introduction

Biofilms of pathogenic bacteria can be formed on the surfaces of patients’ tissues and indwelling medical devices [1,2]. Their structure, bacterial cells protected by extracellular matrix (ECM), determines their intrinsic resistance to both the host effector mechanisms and the action of antibacterial substances [3,4]. Even when a biofilm infection is caused by otherwise drug-sensitive bacteria, the biofilm mode of life increases the tolerance to much higher doses of antibiotics than the same strain in liquid cultures [5,6]. Biofilm infections can be developed on all inner and outer surfaces of the human body. They are considered to be a major cause of chronic infections and, more recently, have been associated with acute infections as well [7,8,9]. Their role in infection, together with their significant drug tolerance, describes the specific role of biofilms as multifactorial virulence factors, having peculiar mechanisms in their relations with the infected host [10]. Not less important are the problems related to biofilm drug tolerance, which represent a serious challenge for the development of novel therapeutic approaches.

One prerequisite for the differences between the infections caused by biofilm-bound or unbound bacteria is the architecture of the sessile communities. Biofilm ECMs are composed of polysaccharides, proteins, extracellular DNA, and small molecules. They can prevent the inwards penetration of host effector molecules and medicines by shielding, binding electrostatically, or otherwise inactivating the antibacterial substances [11]. The physicochemical characteristics of the ECM—high viscosity, electric charge, etc., together with the uneven vertical and/or horizontal distribution of ECM components, create microhabitats with gradients in nutrient and oxygen access within higher and deeper layers of the community [4]. Inside such compartments, the bacterial cells are subjected to and respond to different signals. Hence, biofilm cell sub-populations develop that vary with regards to metabolism, growth rate [4,11], etc. Slow growing and dormant cells situated in the inner layers of the biofilm are less affected, even unresponsive to the action of the antibacterials, no matter whether the bacterial cells may themselves be drug-sensitive or not.

Overcoming the ECM barrier has lately been a serious challenge requiring the development of novel classes of drug-delivery systems [1,12]. Among these, cationic polymer micelles (CPMs) have a good perspective due to their unique structure and small sizes (usually 20–200 nm) [13,14,15]. They are composed of a hydrophobic core surrounded by a positively charged hydrophilic shell. Thus, CPMs are able to accommodate in their core hydrophobic substances such as antibiotics; therefore, they are preferred devices for drug delivery [16,17,18]. On the other hand, the cationic shell provides colloidal stability and protection of the encapsulated drug but also possesses its own antibacterial activity [19,20,21]. CPMs have a number of advantages in drug delivery related to the prolonged blood circulation time, enhanced bioavailability, controlled and targeted drug release, improved pharmacokinetics and biodistribution, capability for overcoming cellular and tissue barriers, etc. A number of cationic polymers have been shown to exhibit antibacterial properties [19,20,21]. Among them, polymeric micelles with a PDMAEMA shell have been shown to significantly reduce the biomass of biofilms by several Gram-positive and Gram-negative species [22]. Their antibiofilm effect was facilitated when they were loaded with silver nanoparticles [23]. However, the usage of polycations is usually accompanied by enhanced cytotoxicity of the drug delivery systems [24,25]. To overcome such problems, recently we proposed a simple strategy involving the preparation of mixed polymer micelles (MPMs) with a shell composed of cationic and non-ionic polymer chains [26]. MPMs of different compositions were prepared by changing the cationic/nonionic components ratio, loaded with ciprofloxacin, and finally to biofilms of *E. coli* and *S. aureus* [26]. The present study investigates the possible application of a PDMAEMA_35_-b-PCL_70_-b-PDMAEMA_35_ and a non-ionic PEO_100_-b-PPO_65_-b-PEO_100_ triblock copolymer as drug-delivery systems for two antibiotics with different modes of action—the quinolone ciprofloxacin (CF) and the macrolide azithromycin (AZ). The antibiotics were loaded in the MPMs at a polymer-to-drug weight ratio of 10. The novelty of this work lies in providing experimental evidence for the capacity of the developed system to disrupt biofilms efficiently, deliver the antibiotics, and sustain the immune system’s reactive and protective functions.

## 2. Materials and Methods

All organic solvents were of analytical grade, purchased from Sigma-Aldrich (Merck-Bulgaria, Sofia, Bulgaria), and used without any further purification. Dichloromethane (DCM) and tetrahydrofuran (THF) used for the synthesis of triblock copolymer were distilled before use after stirring overnight in calcium hydride (95%, Sigma-Aldrich Merck-Bulgaria, Sofia, Bulgaria). Poly(ε-caprolactone) diol (CAPA^®^ 2803, molar mass 8000 g mol^−1^, Solvay Chemicals Inc., Houston, TX, USA) was precipitated in cold methanol (−30 °C), filtered, and then dried overnight under vacuum. 2-(Dimethylamino)ethyl methacrylate monomer (DMAEMA, 98%, Sigma-Aldrich, Merck-Bulgaria, Sofia, Bulgaria) was purified through a column containing activated neutral aluminum oxide (Fluka, FOT, Sofia, Bulgaria) to remove the inhibitors. 2,2′-Azobis(isobutyronitrile) (AIBN, Sigma-Aldrich, Merck-Bulgaria, Sofia, Bulgaria) was used after recrystallization from methanol. 4-cyano-4-(thiobenzoylthio)pentanoic acid, N,N′-dicyclohexylcarbodiimide (DCC, 99%), and 4-dimethylaminopyridine (DMAP, 99%) were received from Sigma-Aldrich (Merck-Bulgaria, Sofia, Bulgaria). Ciprofloxacin (CF, 98%, Acros Organics, Geel, Belgium) was purchased from Fisher Scientific (FOT, Sofia, Bulgaria). Azithromycin (AZ, certified reference material grade) was received from Sigma-Aldrich (Merck-Bulgaria, Sofia, Bulgaria). PEO_100_-b-PPO_65_-b-PEO_100_ (Pluronic F127, molar mass 12,500 g·mol^−1^) was kindly provided by BASF Corporation, Ludwigshafen am Rhein, Germany. For the preparation of micellar dispersions, ultra-pure water (>18 MΩ) was used.

### 2.1. Synthesis of PDMAEMA_35_-b-PCL_70_-b-PDMAEMA_35_ Triblock Copolymer

The cationic PDMAEMA_35_-PCL_70_-PDMAEMA_35_ triblock copolymer was synthesized by reversible addition−fragmentation chain-transfer (RAFT) polymerization as described elsewhere [27]. The experimental details as well as copolymer composition are given in the Supplementary Materials.

Briefly, the synthetic strategy involved: (1) conversion of the terminal hydroxyl groups of PCL-diol into a bifunctional PCL macro chain transfer agent (CTA-PCL-CTA) in the presence of N,N′-dicyclohexylcarbodiimide as a coupling agent and 4-dimethylaminopyridine as a catalyst and (2) polymerization of DMAEMA initiated by CTA-PCL-CTA using AIBN as an initiator. The molecular characteristics of the resulting triblock copolymer are as follows: M_n_ = 18, 900 g mol^−1^, Ɖ = 1.81, polycation content of 58 wt%.

### 2.2. Preparation of MPMs

MPMs were prepared by dissolving appropriate amounts of Pluronic F127 and PDMAEMA_35_-b-PCL_70_-b-PDMAEMA_35_ block copolymers, blended at molar ratios of 3:1, 1:1, and 1:3 in THF, followed by dropwise addition of the organic solution to deionized water. The resulting dispersions were dialyzed against water for 6 days through a dialysis membrane (SpectraPore 7, MWCO 10,000, Repligen, Lund, Sweden) to remove the organic solvent. Single-component polymeric micelles (SCPMs) from Pluronic F127 and PDMAEMA_35_-b-PCL_70_-b-PDMAEMA_35_ block copolymers were prepared for comparative experiments by the same procedure. The final concentration of all micellar aqueous dispersions was 1 mg mL^−1^. They were diluted before usage to give a concentration ranging from 0.5 to 0.125 mg mL^−1^.

### 2.3. Loading of MPMs with Antibiotics

Loading of MPMs with antibiotic (CF or AZ) was performed by addition of an appropriate amount of drug powder to the micellar aqueous dispersion (1 mg mL^−1^) at a polymer-to-drug mass ratio of 10:1. The mixtures were sonicated for 1 h at 60 °C, and then the dispersions were filtered through sterile RC membrane filters with a pore size of 0.2 μm to collect the insoluble drug. Afterward, the filter was rinsed with methanol to dissolve the non-loaded (free) antibiotic, and the fraction was quantified by high-performance liquid chromatography (HPLC). The encapsulation efficiency (EE) and drug loading content (DLC) were determined by the following equations:

$$\mathrm{EE}(\%)=\frac{\left(total\;drug\;added\;amount-free\;drug\;amount\right)}{\left(total\;drug\;added\;amount\right)}\times 100$$

$$\mathrm{DLC}(\%)=\frac{\left(entrapped\;drug\;amount\right)}{\left(micellar\;weight\right)}\times 100$$

### 2.4. Calculation of Solubility and Polymer-Drug Miscibility Parameters

The solubility parameter (δ) for the antibiotics and core-forming hydrophobic polymers was calculated by the group contribution method [28] using the following equation:

$$\delta =\rho \;\frac{\sum Fi}{M},$$

where ρ is the density of the drug or polymer, *M* is the molar mass of drug or polymer, and *F* is the molar attraction constant. For this study, δ was calculated using Hoy molar attraction constants [28].

The polymer-drug miscibility (χ) parameter was determined based on Flory-Huggins theory [29] and calculated by the equation:

$$\chi =\frac{V_{d}}{RT}{\left(\delta_{d}-\delta_{p}\right)}^{2},$$

where *V_d_* is the molar volume of the drug, *δ_d_* and *δ_p_* are the solubility parameters of the drug and core-forming hydrophobic polymer, respectively. *R* is the gas constant, and *T* is the temperature in Kelvin.

### 2.5. In Vitro Release of Antibiotics

The antibiotic (CF or AZ) release was performed in phosphate buffer (pH 7.4). The drug-loaded MPMs (total volume of 3 mL) were placed in dialysis membrane (SpectraPore 7, MWCO 50,000, Repligen, Lund, Sweden), and the membrane was immersed in 30 mL of the dissolution medium at 37 °C. Aliquots of samples were taken from the dissolution medium at specific time intervals, and that volume was replaced with fresh medium to maintain sink conditions. The amount of released drug was determined by HPLC.

### 2.6. Nuclear Magnetic Resonance (NMR) Spectroscopy

^1^H-NMR was used for determination of PDMAEMA_35_-b-PCL_70_-b-PDMAEMA_35_ triblock copolymer molar mass (M_n_). The spectra were recorded using a Bruker Avance-DRX 400 MHz spectrometer (Bruker, Billerica, MA, USA). The samples were dissolved at room temperature in CDCl_3_. The data is available in Supplementary Materials.

### 2.7. Size Exclusion Chromatography (SEC)

The molar mass (M_n_) and dispersity index (Ɖ) of PCL-diol, CTA-PCL-CTA macro-RAFT agent, and PDMAEMA_35_-b-PCL_70_-b-PDMAEMA_35_ triblock copolymer were determined by the Shimadzu Nexera HPLC chromatograph (Shimadzu Coorporation, Kyoto, Japan), equipped with a degasser, a pump, an autosampler, a RI detector, and three PSS SDV columns (5 μm Linear M; 5 μm, 100 Å; and 5 μm, 50 Å). THF as the mobile phase at a flow rate of 1.0 mL min^−1^ and a temperature of 40 °C was used. The concentration of samples was 1 mg mL^−1^, and molecular masses were calculated using polystyrene standards.

### 2.8. High-Performance Liquid Chromatography (HPLC)

HPLC was used for quantification of loaded and released drugs (CF and AZ) using a Shimadzu Nexera 40XR HPLC system (Shimadzu Coorporation, Kyoto, Japan): LC40 solvent delivery up to 700 bar pressure; SIL 40C XR automatic sampler with cooling system; CTO-40S column oven up to 100 °C and SPD-M40 photodiode array detector—scan range 190–800 nm. A gradient elution for 15 min was performed. For CF analysis, an Avantor ACE 5 C18 column (ACE-121-1204—125 mm length and 4.0 mm ID) at 1.0 mL min^−1^ flow rate was used. The mobile phase A contains methanol:buffer solution (25 mM o-phosphoric acid with pH 3.0 and 1 mL Et3N) = 10:90 *v*/*v*, and the mobile phase B contains methanol:buffer solution (25 mM o-phosphoric acid with pH 3.0 and 1 mL Et3N) = 40:60 *v*/*v*. For AZ analysis, an Avantor ACE C18 column (EXL-1217-1546, working at a pH range 2–10—150 mm length and × 4.6 mm ID, Avantor Inc., Radnor, PA, USA) at a 1.0 mL min^−1^ flow rate was used. The mobile phase contains methanol:buffer solution (20 mM KH_2_PO_4_ with pH 8.0) = 10:90 *v*/*v*. All solutions were filtered through 0.2-µm RC-membrane filters before injection, including the mobile phases. A 2 µL CF sample and 5 µL AZ sample were automatically injected for analysis.

### 2.9. Dynamic and Electrophoretic Light Scattering (DLS and ELS)

DLS and ELS were performed on a NanoBrook 90Plus PALS instrument (Brookhaven Instruments Corporation, Nashua, NH, USA), equipped with a 35 mW red diode laser (λ = 640 nm). DLS measurements were carried out at a scattering angle (θ) of 90° and a temperature of 25 °C. The hydrodynamic diameter, D_h_, was calculated using the equation of Stokes–Einstein. The ζ-potential measurements were carried out at a scattering angle (θ) of 15° using ELS and PALS methods. The ζ-potentials were calculated from the obtained electrophoretic mobility at 25 °C by the Smoluchowski equation.

### 2.10. Atomic Force Microscopy (AFM)

The AFM images were obtained using a Bruker NanoScope V9 instrument (Bruker, Billerica, MA, USA) operating at a 1.00 Hz scan rate under ambient conditions. The samples were prepared by spin-coating of a drop of micellar dispersion (2 μL) at 2000 rpm onto a freshly cleaned glass slide. AFM measurements were performed in ScanAsyst mode (Peak Force Tapping, Bruker, Billerica, MA, USA) in air.

### 2.11. Cytotoxicity

We used the human keratinocyte cell line HaCaT for this study. Cells were routinely cultivated in 90 mm petri dishes before experiments and incubated at standard conditions—5% CO_2_, 37 °C, in DMEM, supplemented with 10% FBS and 1% (*v*/*v*) antibiotic–antimycotic solution (penicillin 100 U·mL^−1^, streptomycin 100 μg·mL^−1,^ and amphotericin B 0.25 μg·mL^−1^). Cytotoxicity was estimated through an MTT assay that assesses cell viability as a function of the activity of mitochondrial dehydrogenases of viable cells. All reagents and chemicals were supplied from ThermoFisher Scientific (Waltham, MA, USA) and Sigma-Aldrich (Merck-Bulgaria, Sofia, Bulgaria), unless stated otherwise.

The cells were seeded in 96-well plates at a concentration of 20 × 10^4^ cells per mL for these experiments. They were incubated for 24 h to recover their morphology, cell contacts, and cell cycle after trypsinization. Then the media was carefully removed and replaced with DMEM without antibiotic-antimycotic supplementation and with different concentrations of the polymeric micelles. After treatment for 4 h, the media was discarded and samples were washed with PBS. Cells were incubated with MTT solution (AppliChem, Darmstadt, Germany) until formation of formazan crystals. We used DMSO as a dissolving agent. The optical density of the samples was measured at 570 nm by the Epoch Microplate Spectrophotometer (BioTek, Instruments, Winooski, VT, USA) with the Gen5 Data Analysis software v. 1.9. The results are presented as a percent cell viability compared to the untreated cells, used as a control. The data were analysed with OriginPro 9.0 and presented as a mean ± SD. Statistical significance is according to One-way ANOVA at the *p* < 0.05 level.

### 2.12. Bacterial Strains and Cultivation

The following strains of *E. coli* were included in the study: *E. coli* 25922 (ATCC) and several UPEC strains: the serotype-specific reference strains O4:K32:H5 (SS) and O18:K32:H5 (SS), kindly provided by Dr. R. Ivanova, Bulbio-NCIPD, and the clinical isolates PU-1, PU-3, PU-13, and PU-19. The biofilm-forming capability of the UPEC strains has been previously established [30,31]. *P. aeruginosa* was represented by strains from the International Reference panel [32]: PAO1 (ATCC 15692)—a widely studied wound isolate, as well as the following isolates: PA14 (human burn isolate), two keratitis isolates (39016 and 39177), and A5803—a strain from community-acquired pneumonia. Strain maintenance was as described previously [26], and overnight cultures in Trypticase soy broth TSB) (Himedia, Maharashtra, India) at 37 °C were used as inoculum. Biofilm experiments were performed in minimal salt medium M63 (0.02 M KH_2_PO_4_, 0.04 M K_2_HPO_4_, 0.02 M (NH_4_)_2_SO_4_, 0.1 mM MgSO_4_, and 0.04 M glucose, pH 7.5).

### 2.13. Biofilm Dispersion Experiments

The overnight inoculum broth culture was suspended and diluted 1:100 in M63 medium. The bacterial suspensions were applied onto 96-well polystyrene U-bottom plates (Nunc, ThermoFisher Scientific, Waltham, MA, USA) to cultivate 24 h biofilms, as described earlier [26]. After triple washing of the developed biofilms with PBS, the polymeric micelles, empty or loaded with ciprofloxacin (CF) or azithromycin (AZ), were applied onto the biofilm in concentrations of 0.5, 0.25, or 0.125 mg mL^−1^. On each plate, one column of wells with developed biofilm was washed with PBS and then left without medium and was further used for measurement of the initial, pre-treatment biofilm (0 h-control). The treatments were performed for 4 or 24 h at 37 °C, followed by the removal of the micelles, washing, and crystal violet (CV) staining of the biofilms. Each variant was repeated in 6 wells. The data was calculated and are presented as a percentage of the initial biofilm (0 h control).

### 2.14. Biofilm Cell Viability Estimation

Biofilms were cultivated for 24 h as described above. After washing with triple changes of PBS, 150 µL of the micelles 3:1, 1:1, and 1:3, either empty or loaded with CF or AZ, were applied in a concentration of 0.25 mg mL^−1^. At the start of the treatment, the resazurin-based Alamar Blue Cell Viability Reagent (Invitrogen, Thermo Fisher Scientific, Waltham, MA, USA) was added to each well to reach a final concentration of 1%. Each variant was repeated in 6 wells. Controls included wells containing only dH_2_O (the medium for micelle dispersion) and wells filled with M63 medium (designated as “untreated control”). After 24 h of incubation, the absorbance was measured at wavelengths of 570 and 620 nm. The viability of the bacterial cells was calculated as a percentage of the untreated control (the absorbance values at λ_1_ = 570 nm and λ_2_ = 620 nm of the biofilm in M63 medium) according to the following formula:

$$Viability\;\left(\%\right)rel\;to\;M63\;=\frac{\left[\left(\varepsilon ox\right)\lambda_{2}A\lambda_{1}-\left(\varepsilon ox\right)\lambda_{2}A\lambda_{2}\right]}{\left[\left(\varepsilon ox\right)\lambda_{2}Ao\lambda_{1}-\left(\varepsilon ox\right)\lambda_{1}Ao\lambda_{2}\right]}\times 100$$

where Aλ_1_ and Aλ_2_ were the absorbance values measured for the treated wells at λ_1_ = 570 nm and λ_2_ = 620 nm, and Aoλ_1_ and Aoλ_2_ were the average (of 6 repeats) of the absorbances measured for the M63 control. The values of the extinction molar coefficients of the Alamar Blue reagent at the respective wavelengths, i.e., (εox)570 = 80.586 and (εox)600 = 117.216, were as provided by the manufacturer.

### 2.15. Scanning Electron Microscopy (SEM)

Polystyrene pieces, 0.5 × 0.5 cm, cleaned with detergent followed by 5 min ultrasonication in 70% ethanol and UV sterilized, were used as the substratum for biofilm cultivation. The pieces were placed in the wells of 24-well plates, covered with bacterial suspensions in M63 medium prepared as above, and the biofilms were cultivated for 24 h at 37 °C. Then the samples were washed and treated with 0.25 mg/m^−1^ of empty or CF- or AZ-loaded micelles, similarly to the above-outlined protocol applied to the 96-well trials. Treatment was performed for 24 h; the samples were washed in 0.2 M Na-cacodylate buffer, pH 7.4, and fixed for 2 h with 4% cacodylate-buffered glutaraldehyde. Followed by 1 h post-fixation in 1% cacodylate-buffered OsO_4_, dehydration in a graded ethanol series, and mounting on sample holders. After sputter-coating with gold, the observations were made on the Lyra\Tescan electron microscope (Tescan Analytics, Brno, Czech Republic) at an accelerating voltage of 20 kV.

### 2.16. Preparation of Murine Skin Explants

Male or female mice from the outbred ICR strain were purchased from the Slivnitsa Animal Farm of the Bulgarian Academy of Science (Slivnitsa, Bulgaria). Animals were housed in standard cages (5 per cage) with a wood chip bedding and under the standard conditions. The animals had free access to tap water and food (containing 29% protein, 13% fat, and 56% carbohydrates) and daily health monitoring. All experiments were performed under veterinary supervision and according to national legislation and guidelines (SG Issue 87/2006) and Decree 20/01.11.2012; License for Animal Housing #352/30.01.2012 (#11130005); License for Experimental Procedures #125/07.10.2020. All experiments were conducted in accordance with the ARRIVE criteria (Animal Research: Reporting of In Vivo Experiments) and the principles of the 3Rs (Replacement, Reduction, and Refinement).

Skin explants were prepared aseptically from the dorsal back skin of 5 ICR mice, 10 weeks-old, weighing 22 ± 1 g. The skin was collected from each mouse after hair removal (hair removal cream, 10 min treatment), disinfection with 70% ethanol, followed by 2 washes with sterile dH_2_O. Skin samples with a size of 3 × 3 cm^2^ were placed in a sterile petri dish (Costar, Göttingen, Germany) and cut with a sterile blade to a size of 1 × 1 cm^2^. Each skin sample was placed into the bottom of a 12- or 24-well plate (TPP, Trasadingen Switzerland) and cultured as an air–liquid interface explant in pre-warmed 250 µL/well Dulbecco’s Modified Eagle’s Medium (DMEM) containing 4500 mg L^1^ glucose, stable glutamine, sodium bicarbonate, sodium pyruvate (Sigma-Aldrich, Merck-Bulgaria, Sofia, Bulgaria), and 10% bovine calf serum (FBS; Sigma-Aldrich, Merck-Bulgaria, Sofia, Bulgaria).

### 2.17. P. aeruginosa Biofilm Model on Ex Vivo Murine Skin Explants

The ex vivo skin explant model was adapted from the method of Phillips et al. [33]. The skin explants were infected with 25 µL of *P. aeruginosa* PAO1 suspension to form biofilm for 24 h. Then they were washed with sterile dH_2_O and treated with 50 µL of the following solutions: MPM 1:1 loaded with CF or AZ, with final concentrations of the MPMs of 0.5 or 0.25 mg mL^−1^. Controls comprised samples treated with dH_2_O (the medium in which the MPMs were dispersed) and PBS. Samples of non-infected explants treated similarly to infected were also used. The plate with uninfected and biofilm-infected skin explants was centrifuged after 24 h at 250× *g*, 5 minute, and the supernatants were aseptically collected, passed through a sterile syringe and 20 µm-pore filters (Millipore, Merck, Darmstadt, Germany), aliquoted, and frozen at −20 °C and used in analyses of nitric oxide and IL-17A secretion. The presence of *P. aeruginosa* infection was confirmed by plating on Cetrimide agar (Sigma-Aldrich, Merck-Bulgaria, Sofia, Bulgaria).

### 2.18. Determination of NO Production by Ex Vivo Skin Explants

Nitric oxide (NO) production by skin explants was determined with a colorimetric Griess reaction. Prior to analysis, the supernatant samples were thawed at 37 °C and then deproteinized using the 10 kDa Spin-X^®^ UF concentrator (Sigma-Aldrich, Merck-Bulgaria, Sofia, Bulgaria) and centrifugation at 10,000× *g*, 4 °C for 10 min. The filtrate was collected, diluted 1:5 with PBS, and 100 µL of each sample were added to a flat-bottom plate (Costar, Göttingen, Germany). The reaction was performed by adding 100 µL of Griess reagent (0.2% naphthylethylenediamine dihydrochloride (NEDD), 2% sulfanilamide in 5% phosphoric acid, all from Sigma-Aldrich, Merck-Bulgaria, Sofia, Bulgaria). This reagent reacts with nitrite in the samples to form a purple azo product. The reaction was allowed to develop for 30 min in the dark, at room temperature. The absorption was measured by an EL800 microplate reader (BioTek) at 550 nm. The concentration of NO was determined from a standard curve of NaNO_2_. The concentration of nitrite (in mM) in the test samples was calculated according to the formula:

$$Nitrite\;concentration=\left(\frac{B}{V}\right)*D$$

where *B* is the amount of nitrite in the sample calculated from the standard curve in (nmoL), *V* is the sample volume added in the sample wells (mL), and *D* is the sample dilution factor.

### 2.19. Secretion of IL-17A by Skin Explants

IL-17 production by the skin explants and its secretion in the cultures was determined with an ELISA kit according to the manufacturer’s protocol (#900-K392, the sensitivity range of 16–1000 pg/mL^−1^; Peprotech, Cranburry, NJ, USA). The MaxiSorp™ flat-bottom 96-plate (#442404, Nunc-Immuno™, Thermo Fisher Scientific, Waltham, MA, USA) was pre-coated with 1.0 μg/mL^−1^ capture antibody in PBS (100 µL/well) for 24 h, at 4 °C. The plate was washed 3 times with wash buffer containing 0.05% Tween-20 (#P-7949, Sigma-Aldrich) in PBS (300 µL/well). Then, the plate was blocked with buffer containing 1% BSA (#A-7030; Sigma-Aldrich) in wash buffer (200 μL/well) for 2 h at room temperature by shaking on a mini-rocker (Bio-Rad, AA Medical Bulgaria Ltd., Sofia, Bulgaria). The plate was washed 4 times with the wash buffer (300 μL/well) by shaking on a mini-rocker for 3 minutes each time. The supernatant’s samples were thawed at 4 °C for 1 h and then spun down on a benchtop minicentrifuge at 500× *g*, 1 min. The supernatants were diluted 1:5, and the standards were diluted from 0–1000 pg/mL^−1^ in 0.05% Tween-20/0.1% BSA/PBS and were added (100 µL/well) in triplicates to the plate. The plate was incubated overnight at 4 °C, then washed 3 times with wash buffer, and 0.25 µg/mL^−1^ of the biotinylated rabbit anti-murine IL-17A detection antibody (100 μL/well) was added for 2 h at room temperature. The plate was washed 3 times and incubated with avidin-HRP conjugate (1:2000 diluted in 0.05% Tween-20/0.1% BSA/PBS) (100 μL/well) for 30 minutes at room temperature. After washing 4 times, ABTS (2,2′-Azino-bis(3-ethylbenzothiazoline-6-sulfonic acid) Liquid Substrate Solution (#A3219; Sigma-Aldrich, Merck-Bulgaria, Sofia, Bulgaria) (100 µL/well) was added and incubated in the dark until the reaction developed. The absorption at 405 nm with a wavelength correction at 650 nm was measured on an Innova microplate reader (Infitek, Spokane, WA, USA). The concentration of the cytokine in the samples was calculated from a standard curve of recombinant mouse IL-17A.

### 2.20. Histological Evaluation of Skin Explants

The ex vivo skin explants with *P. aeruginosa* biofilm were treated as described above, washed 3 times with sterile PBS, and were fixed with 10% formaldehyde/PBS solution (Sigma-Aldrich, Merck-Bulgaria, Sofia, Bulgaria). Following fixation, the tissues were dehydrated in increasing concentrations of ethanol (70%, 80%, 96%, and 100%), after which they were incubated in the histological clearing agent Histo-Clear II (National Diagnostics, Atlanta, GA, USA) and embedded into paraffin (Paraplast, Sigma-Aldrich, Merck-Bulgaria, Sofia, Bulgaria). Tissue blocks were sectioned at a thickness of 5 µm on an Accu-Cut SRM 200 rotary microtome (Sakura Finetek, Antisel Bulgaria, Sofia, Bulgaria). The sections were mounted onto gelatin-coated glass slides and allowed to air-dry.

H and E staining was performed according to the standard protocol. Briefly, the sections were cleared of paraffin using two changes of Histo-Clear II and then re-hydrated sequentially in absolute, 96% and 70% ethanol. The sections were stained for 10 min with Mayer hematoxylin, washed with warm tap water, then rinsed in distilled water and briefly dipped in 96% ethanol. The sections were counter-stained in Eosin Y solution, then dehydrated through 96% and absolute ethanol, and, finally, cleared with two changes of Histo-Clear II. The slides were mounted with xylene-based mounting medium DePeX (Serva, Heidelberg, Germany). The stained tissue sections were observed on a Leica DM2000 microscope using the Leica Application Suite X (LAS X) software v. 3.7.4.23463, and images were captured with a Leica ICC50 W microscope camera at 40× and 63× magnification.

## 3. Results and Discussion

### 3.1. Preparation and Characterization of MPMs

MPMs were formed at three molar ratios (3:1, 1:1, and 1:3) by dropwise addition of blended copolymer organic solution to aqueous media followed by dialysis against water. All dispersions were prepared at a concentration of 1 mg mL^−1^, and then diluted with water to concentrations of 0.5 and 0.125 mg mL^−1^.

**Figure 1:**
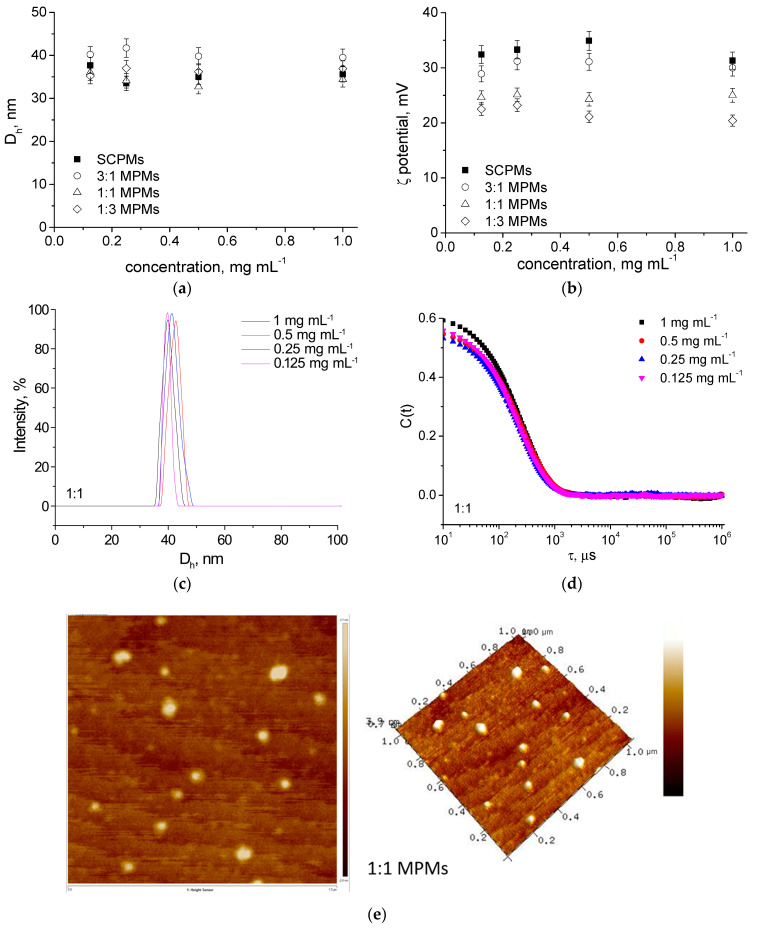
(**a**) Hydrodynamic diameter, D_h_, and (**b**) ζ-potential variations as a function of the micellar concentration of SCPMs and MPMs based on PDMAEMA_35_-PCL_70_-PDMAEMA_35_ and Pluronic F127 triblock copolymers. (**c**) Size distribution curves (**d**) DLS correlation functions and (**e**) representative AFM micrograph of MPMs prepared from PDMAEMA_35_-PCL_70_-PDMAEMA_35_ and Pluronic F127 triblock copolymers at a molar ratio of 1:1 in the concentration range of 1 to 0.125 mg mL^−1^. The PDI values ranged in the 0.11–0.19 interval. All DLS measurements were performed at 25 °C.

DLS and ELS were used to characterize the resulting particles. In consistency with previous results [26], MPMs possessed a hydrodynamic diameter ranging from 31 to 43 nm, a monomodal size distribution, and a positive ζ-potential, which was dependent on their composition (Figure 1a–c). As evident from Figure 1a–d, the physicochemical characteristics of the MPMs were not influenced by the concentration of the micellar solution as the D_h_ and surface charge remained unchanged after dilution. It has to be mentioned that the lowest concentration of 0.125 mg mL^−1^ was identical (or lower) to the critical solution concentration (CMC) of micellar compositions 1:1 and 1:3 (0.124 and 0.131 mg mL^−1^, respectively; see Figure S4). Nevertheless, these micelles remain stable upon dilution due to the stabilizing effect of the PCL core provoked by the semi-crystalline nature of this polymer [26]. The stability of the particles was well demonstrated by the correlation functions of the MPMs, which preserved their characteristic sigmoidal curve pattern at the whole range of concentrations (Figure 1d). In contrast, multiple scattering occurred for F127 due to structure disintegration even at a concentration of 0.5 mg mL^−1^ (see Supplementary Materials).

The morphology of the MPMs was visualized by AFM. As evident from Figure 1e, the particles were characterized by spherical morphology and a size of about 32 nm. This result is in good agreement with the data from DLS measurements.

### 3.2. Loading of MPMs with Antibiotics and Release

**Figure 2:**
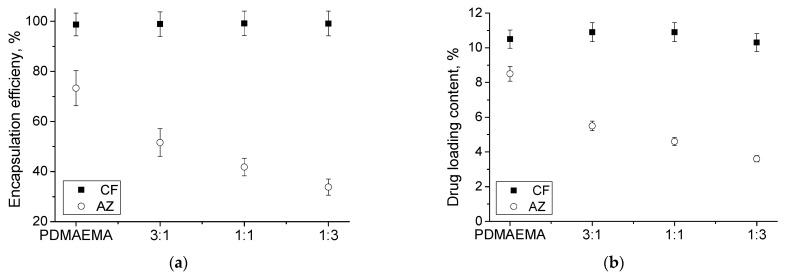
Variations of encapsulation efficiency (**a**) and drug loading content (**b**) as a function of the composition of SCPMs and MPMs based on PDMAEMA_35_-PCL_70_-PDMAEMA_35_ and Pluronic F127 triblock copolymers. The loading was performed at polymer-to-drug mass ratio of 10:1.

The formed MPMs were loaded with the antibiotics CF or AZ, which were selected because they are wide-spectrum antibiotics approved by the Food and Drug Administration (FDA) against various bacterial infections. Additionally, in a previous study, a series of experiments focused on the loading of CF in MPMs at various polymer-to-drug mass ratios were performed, and at optimal ratios, a very high (90–98%) encapsulation efficiency (EE) was observed [26]. Based on this experience, in the current study the antibiotics were loaded into the micelles at a polymer-to-drug mass ratio of 10:1, and the effect of drug nature was investigated. The loading was performed by sonicating MPMs and the drug powder for 1 h at 60 °C. Then the dispersions were filtered to collect the insoluble drug fraction. The EE and DLC were determined by quantifying the filter fractions by HPLC. The corresponding concentration of antibiotics is summarized in Table S3. The variations of the two parameters with the micellar composition are shown in Figure 2. It could be noted that the EE and DLC values of CF for all the systems were very high (closed symbols). The enhanced EE and DLC values in the CF-loaded systems are probably due to the fact that drug molecules are localized not only in the micellar core but also in the shell [26]. The latter is achieved as a consequence of an electrostatic interaction of positively charged PDMAEMA with the zwitterionic CF, as previously described. In contrast, the loading of AZ (open symbols) was strongly influenced by the micellar composition. EE and DLC decreased with increasing the PPO content in the micellar core. The low compatibility of AZ and PPO was further confirmed by determining the polymer-to-drug miscibility (χ) parameter using the Flory-Huggins theory [29]. Firstly, using the group contribution method [28], the solubility parameter (δ) for the antibiotics and core-forming hydrophobic polymers was calculated. Then, the compatibility of each couple was assessed by calculating χ. The resulting values of δ and χ are summarized in Table 1. According to the Flory-Huggins theory, AZ has excellent miscibility with the PCL block (χ_AZ_ = 0.03), since the closer the χ value is to 0, the higher the compatibility of the two components [28,29]. Concerning the PPO block, a higher value of χ_AZ_ (2.2) revealed the lower compatibility of AZ and the polyether. Concerning the CF, it could be seen that the miscibility is lower with both PCL and PPO (χ_CF_ = 1.82 and 5.34), meaning that the loading of this antibiotic is performed predominantly in the micellar corona.

**Figure 3:**
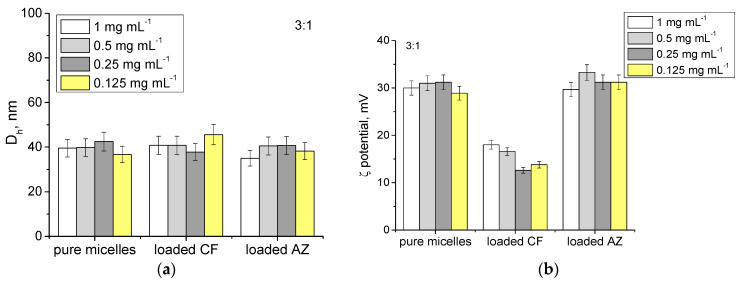

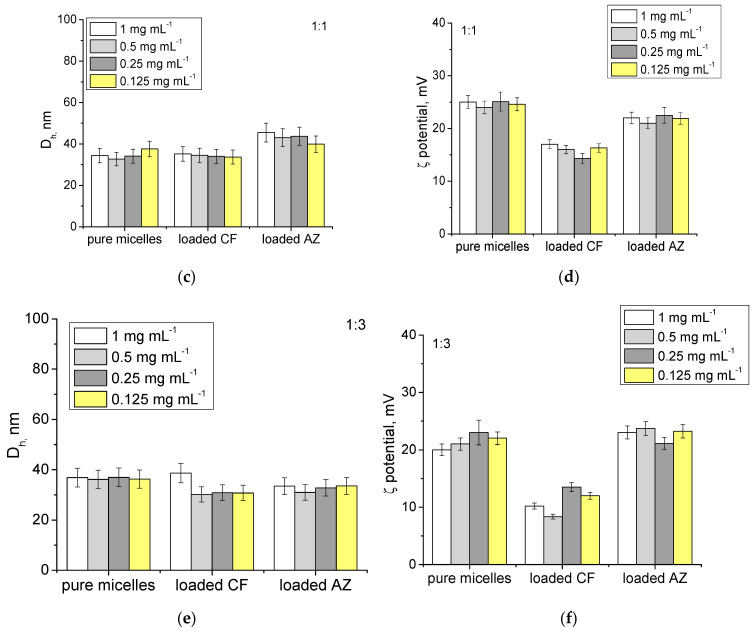
Hydrodynamic diameter, Dh, (**a**,**c**,**e**) and ζ potential (**b**,**d**,**f**) of empty or loaded with antibiotics MPMs based on PDMAEMA_35_-PCL_70_-PDMAEMA_35_ and Pluronic F127 triblock copolymers in the concentration range of 1 to 0.125 mg mL^−1^. Measurements were performed at 25 °C at pH 7. Each data point represents the arithmetic mean ± SD of three separate experiments.

The filtered aqueous dispersions were analyzed by dynamic and electrophoretic light scattering. The hydrodynamic diameter and ζ-potential of the drug-loaded MPMs of different concentrations were determined (Figure 3). It was observed that neither the concentration nor the type of antibiotic significantly influenced the particle size (Figure 3a,c,e). In contrast, the ζ-potential varied depending on the antibiotic loaded into the MPMs. The loading of CF in the micellar corona through electrostatic interactions with the PDMAEMA block resulted in a neutralization of positive charges followed by a reduction of the ζ-potential values (Figure 3b,d,f). On the other hand, the excellent compatibility of AZ with the PCL block ensured its localization in the micellar core without influencing the particles’ surface charge.

**Figure 4:**
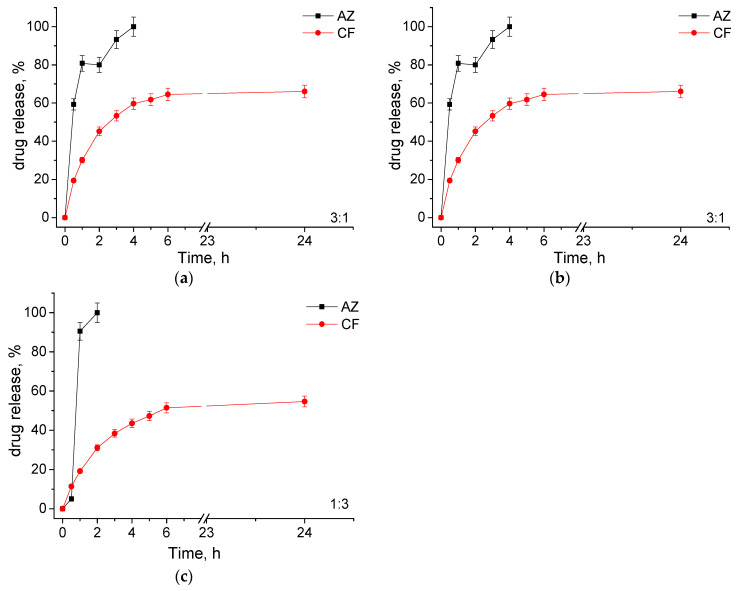
Drug release profiles of MPMs based on PDMAEMA_35_-PCL_70_-PDMAEMA_35_ and Pluronic F127 triblock copolymers, prepared at a 10:1 polymer-to-drug mass ratio, determined by HPLC. MPMs were formed at molar ratios of 3:1 (**a**), 1:1 (**b**), and 1:3 (**c**). The release was performed at 37 °C in phosphate buffer pH 7.4. Each data point represents the arithmetic mean ± SD of three separate experiments.

The antibiotic release from MPMs was investigated in phosphate buffer (pH 7.4) at physiological temperature. These conditions were selected to resemble the extracellular fluid pH. The amount of released drugs was determined by HPLC. Representative chromatograms are given in the SI. The release profiles of the two antibiotics are shown in Figure 4. Generally, the CF-loaded systems were characterized by a prolonged release profile, while the AZ-loaded MPMs exhibited a burst release. The effect of micellar composition on the release profile was negligible for CF-loaded particles and well pronounced at AZ-loaded micelles. It is noticed that the higher the fraction of Pluronic 127 in the particles’ composition, the faster the release of AZ. Apparently, this behavior is related to the miscibility of AZ with the two polymers forming the micellar core. In particular, the higher affinity of AZ to the PCL block is responsible for the delayed drug release, since the 3:1 composition released the total drug amount for 4 h while the 1:3 released AZ only for 2 h. In addition, it has to be mentioned that at SCPMs formed from the PDMAEMA_35_-PCL_70_-PDMAEMA_35_ copolymer, a sustained release up to the end of the investigated time period was observed (Figure S7). In contrast, the prolonged release profiles of CF were related to the electrostatic interactions with the PDMAEMA block and the drug loaded predominantly in the micellar corona. As observed previously, due to the sensitivity of PDMAEMA to changes in pH and temperature, under the release conditions (pH 7.4 and 37 °C), the deprotonation of amino groups causes repulsion of CF molecules from the complex [26].

### 3.3. In Vitro Cytotoxicity

**Figure 5:**
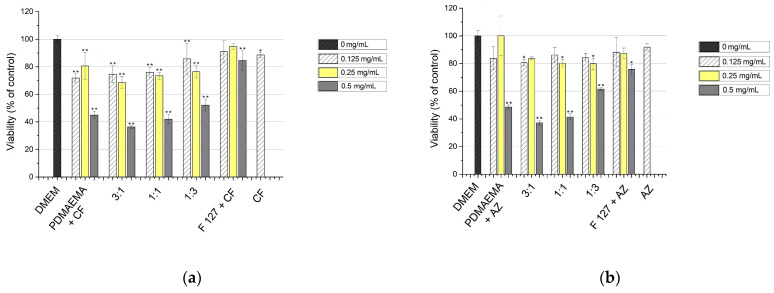
Cytotoxicity of the SCPMs and MPMs based on PDMAEMA_35_-PCL_70_-PDMAEMA_35_ and Pluronic F127 triblock copolymers loaded with CF (**a**) or AZ (**b**) at a 10:1 polymer-to-drug mass ratio. The micelles were applied for 4 h in concentrations of 0.5, 0.25, and 0.125 mg mL^−1^ onto confluent cultured HaCaT. The results are presented as percentage of the control—cells cultivated parallelly in DMEM. The data are the means of four repeats and are presented as the mean ± SD. Differences between control (DMEM) and treated with micelles cells are accepted as statistically significant (*) when *p* < 0.05 and (**) when *p* < 0.001.

In a previous study, we have shown the effectiveness of the MPMs examined here, applied in 1 mg mL^−1^, unloaded or loaded with CF, against developed 24 h biofilms of *E. coli* and *S. aureus* [26]. It was also shown that the addition of Pluronic F127 in the structure of MPMs reduced the micellar cytotoxicity proportionally to the amount of nonionic component. However, when the micelles were loaded with CF, their cytotoxicity was increased, and they reduced the viability of cultured human skin fibroblast cells [26]. One of the easiest ways to overcome this effect could be by lowering the micellar concentration, as also shown previously for silver-loaded PDMAEMA-based micelles [23]. Therefore, the aim of the first series of biological experiments in the present study was to find out the appropriate concentrations of the micelles loaded with CF or AZ that would have acceptable in vitro cytotoxicity and could be used safely in practice. Based on the data obtained from the drug-release profile (a maximum release within 4 to 6 h depending on the antibiotic), the cytotoxicity experiments were performed within a 4 h treatment with the MPM. Figure 5 illustrates that the drug-loaded micelles applied as 0.5 mg mL^−1^ were still toxic, excluding pure Pluronic F127 and triblock copolymers with a prevalence of Pluronic in a proportion of 1:3, while lower concentrations such as 0.25 and 0.125 mg mL^−1^ were with significantly reduced toxicity, and the viability of the treated HaCaT cell cultures was over 70% in all variants (Figure 5a). The comparison of the results between the experiments with MPMs loaded with the different antibiotics showed that the polymeric micelles in combination with AZ had lower toxicity at the two lower concentrations, 0.25 and 0.125 mg mL^−1^, compared to the same samples with CF (Figure 5a,b). The antibiotics themselves, when applied in amounts relevant to their presence in the MPMs (0.125 mg mL^−1^), did not affect the viability of the cultured cells (Figure 5). However, the antibiotics themselves, when not included in MPMs, had no effect on either biofilm reduction or the viability of biofilm bacterial cells [26].

### 3.4. Effects of the Mixed Micelles on Pre-Formed 24 h Biofilms

**Figure 6:**
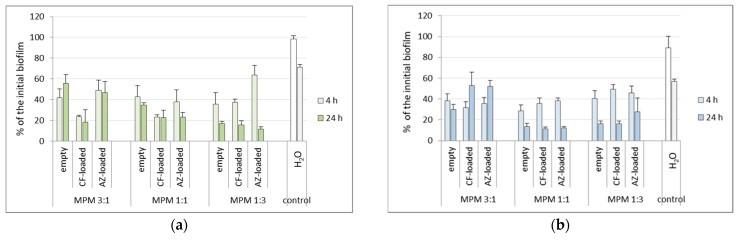
Reduction of the biomass of mature 24 h biofilms as a result of treatment for 4 or 24 h with 0.25 mg mL^−1^ of empty or antibiotics-loaded MPMs based on PDMAEMA_35_-PCL_70_-PDMAEMA_35_ and Pluronic F127 triblock copolymers. The results were calculated as percentage of the biofilm at the start of each experiment. (**a**) *E. coli* 25922; (**b**) *P. aeruginosa* PAO1. Results for biofilms treated with dH_2_O are included since the micelles were dispersed in dH_2_O. Each data point represents the mean ± SD of six repeats.

The next objective was to optimize the conditions for the MPMs application; hence, an appropriate combination between biofilm destruction and low cytotoxicity was sought. The concentration-dependent antifouling effects of the MPMs were examined during 4 and 24 h and were calculated as percentage of the initial biofilm (average biofilm amount of “zero-control” wells estimated by CV coloring of the biofilms developed at time point “0”, i.e., before the start of the treatment). The comparisons of the effects of the 0.5, 0.25, and 0.125 mg mL^−1^ concentrations (Supplementary Figures S8 and S9), together with the trends of biofilm change between the two checked time points, pointed out the 0.25 mg mL^−1^ concentration of all micelles as the optimal one, combining low cytotoxicity (see Figure 5) with good exfoliating effect. The data on biofilm reduction within the two tested time points upon treatment with the 0.25 mg mL^−1^ concentrations of the micelles are shown in Figure 6.

We next asked whether the application of the antibiotic-loaded micelles resulted in successful drug delivery. We first analyzed the biofilm-reduction data by ANOVA-based comparison between the biofilms treated with empty vs. antibiotic-loaded micelles (data for 24 h post-treatment is included in Tables S4 and S5). For *E. coli* 25922, statistically significant differences between empty and drug-loaded micelles implied that the composition 3:1 was most appropriate for drug delivery. The latter could be related to the enhanced amount of PDMAEMA_35_-PCL_70_-PDMAEMA_35_ triblock copolymer in this composition exhibiting a higher EE for the two antibiotics (Figure 2). Moreover, at this composition the release was more sustained regarding the two drugs (see Figure 4). For *P. aeruginosa* PAO1, statistics pointed out the 1:1 MPMs as the most successful in drug delivery.

**Figure 7:**
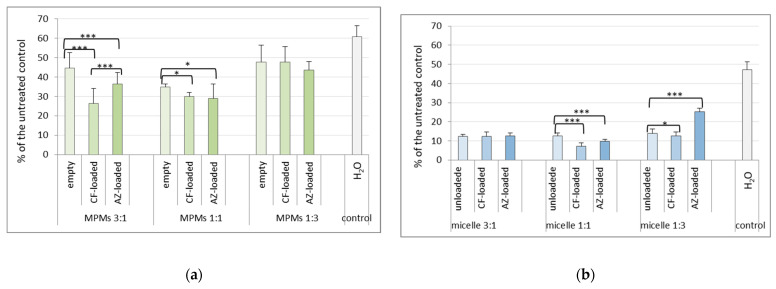
Viability of the biofilms after treatment for 24 h with 0.25 mg mL^−1^ of empty or antibiotics-loaded MPMs based on PDMAEMA_35_-PCL_70_-PDMAEMA_35_ and Pluronic F127 triblock copolymers. Viability was estimated by the reduction of resazurin using the Alamar Blue reagent (Invitrogen). The results were calculated as percentage of the untreated control (biofilm cultivated parallelly in M63 medium in the absence of the tested agents). dH_2_O bars are included to show the effect of treatment with dH_2_O alone_ the medium in which the micelles were dispersed. (**a**) *E. coli* 25922; (**b**) *P. aeruginosa* PAO1. Each data point represents the mean ± SD of six repeats. *p* < 0.05 (*); *p* < 0.001 (***), ANOVA test.

The CV method for biofilm analysis is a methodology most widely-applied in antibiofilm studies; however, it is incapable of discriminating between living cells and dead bacteria that may remain incorporated in the ECM. Therefore, the combined application of more than one method becomes essential in testing novel antibiofilm agents [34]. The following step was to clarify whether and how the treatments influenced the viability of the biofilm bacterial cells. To answer this, a method based on resazurin reduction was applied (using the Alamar Blue Bacterial Viability Kit, Invitrogen). Previous studies have shown that this method is reliable for bacterial viability studies, with good correspondence between the fluorometric data and the parallel determination of colony-forming units [9,35]. Drug delivery should be accompanied by a statistically significant reduction of the viability percenaget between the antibiotic-loaded and the empty micelles. As is shown in Figure 7a, for *E. coli* 25922, the Alamar Blue tests showed that the highest was the viability reduction of loaded compared to empty MPMs 3:1, followed by MPMs 1:1. For *P. aeruginosa* PAO1, the best result was with MPMs 1:1 (Figure 7b). This was followed by the CF-loaded MPMs 1:3. However, unlike the CF-loaded, the AZ-loaded MPMs 1:3 increased instead of lowering the viability of the PAO1 biofilm, which was in accordance with the result from the CV assay (see Table S5).

If the data for biofilm destruction are taken together with the viability of the remaining bacterial cells, it becomes obvious that there is a difference in the effectiveness of the different MPMs when applied to the two bacterial species. Biofilm biomass reduction could be observed to some extent or another with all three types of micelles. However, the data for significant differences between the empty and loaded MPMs on bacterial viability implied that putative drug delivery was performed with different effectiveness in the two model biofilms. Thus, it appeared that the MPMs 3:1 were most successful for *E. coli*, and for *P. aeruginosa,* MPMs 1:1 should be recommended. Due to the cytotoxicity data for the micelles at a concentration of 0.5 mg mL^−1^, and the closer values of CMC to the concentration of 0.125 mg mL^−1^, MPMs concentration of 0.25 mg mL^−1^ was accepted as the most appropriate and was used in the following experiments.

**Figure 8:**
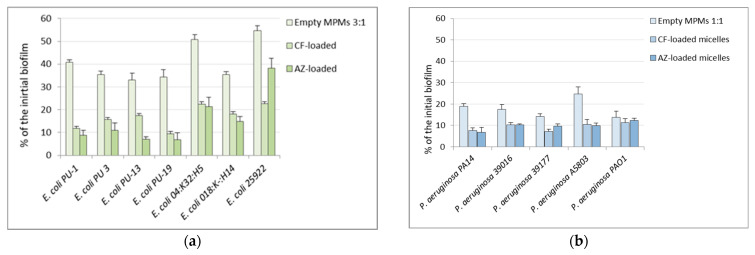
Reduction of biofilms of pathogenic strains of *E. coli* treated with empty or antibiotic-loaded MPMs 3:1 (**a**) and of *P. aeruginosa* treated with empty or antibiotic-loaded MPMs 1:1 (**b**). The results were calculated as percentage of the “0” controls, i.e., the amount of biofilms of the strains before the start of the treatments. Each data point represents the mean ± SD of six repeats.

Next, the effects of the thus-selected micellar compositions were applied on several clinical isolates of the two Gram-negative bacterial species. Together with some strain-specific effects, the results showed good exfoliation effects on all tested 24 h biofilms of clinical strains. The promising result was that when the drug-loaded micelles were used, the biofilm destruction in all tested strains was higher (Figure 8). It should also be pointed out that the biofilm reduction of the *P. aeruginosa* strains was stronger compared to the tested *E. coli* strains.

The results from both the CV assay and the viability tests indicated that the effects of the three types of micelles differed between the two bacterial species as well as between the laboratory and clinical strains included in the study. These differences could be determined by the combination of several factors. On their way to the bacterial cells, the micelles have to overcome the barrier of the biofilm extracellular matrix (ECM). Their interactions with it could be related to both the physicochemical characteristics (D_h_ and ζ potential) and, on the other hand, with the peculiarities of the extracellular matrices of the two types of biofilm [36]. The latter contain polysaccharides, extracellular DNA, proteins, and small molecules [37,38].

Most abundant is the data on the polysaccharide components of ECMs. It is known that *E. coli* releases poly-N-acetyl D-glucosamine and colanic acid (a heteropolysaccharide composed of repeating units containing glucose, fucose, galactose, and glucuronic acid), and some strains are also capable of producing cellulose composed of β-linked D-glucose units [12,31,39,40]. The ECM of *P. aeruginosa* may contain the polysaccharides alginate, Pel, and Psl [41]. Alginate is a heteropolysaccharide composed of 1,4-linked subunits of β-D-mannuronic acid and α-L-glucuronic acid [42]. The repeating unit of Pel is a partially acetylated 1–4 glycosidically linked N-acetyl galactosamine and N-acetyl glucosamine, and Psl contains repeating pentamers composed of D-mannose, L-rhamnose, and D-glucose [43]. The polysaccharides in the ECM may be neutral or charged. Alginate and colanic acid are polyanionic, while poly-N-acetyl glucosamine and Pel are polycationic [38,40,44]. The relative amounts of the individual components may create differences in the total biofilm charge or create differently charged microenvironments. All this can influence the penetrability of antibacterials and the realization of the drug delivery [2,36].

The knowledge on ECM is still insufficient and represents an important future focus that would help to understand the virulence characteristics of bacterial biofilms. A more precise idea of the ECM structure would also facilitate the development of antibiofilm approaches directed to the destruction of the ECM of clinically relevant bacteria [1,45]. For the bacterial species included in this study, varieties of both the proportions of the individual components and their spatial organization within the biofilm could be decisive for the interactions of the ECM with the nanoparticles. Currently, the data in this regard is scarce. Earlier fluorescence microscopy methodologies based on lectin binding [46,47,48,49] or the development of pH-sensitive fluorescent probes [50] have brought forward the notion that the ECM is heterogeneous and may undergo dynamic changes. ECM components of *P. aeruginosa* biofilms were shown to be recognized by the innate immunity via the host C-type lectins [49]. However, this recognition might not be effective because of the ECM thickness that prevents access to the bacterial cells.

**Figure 9:**
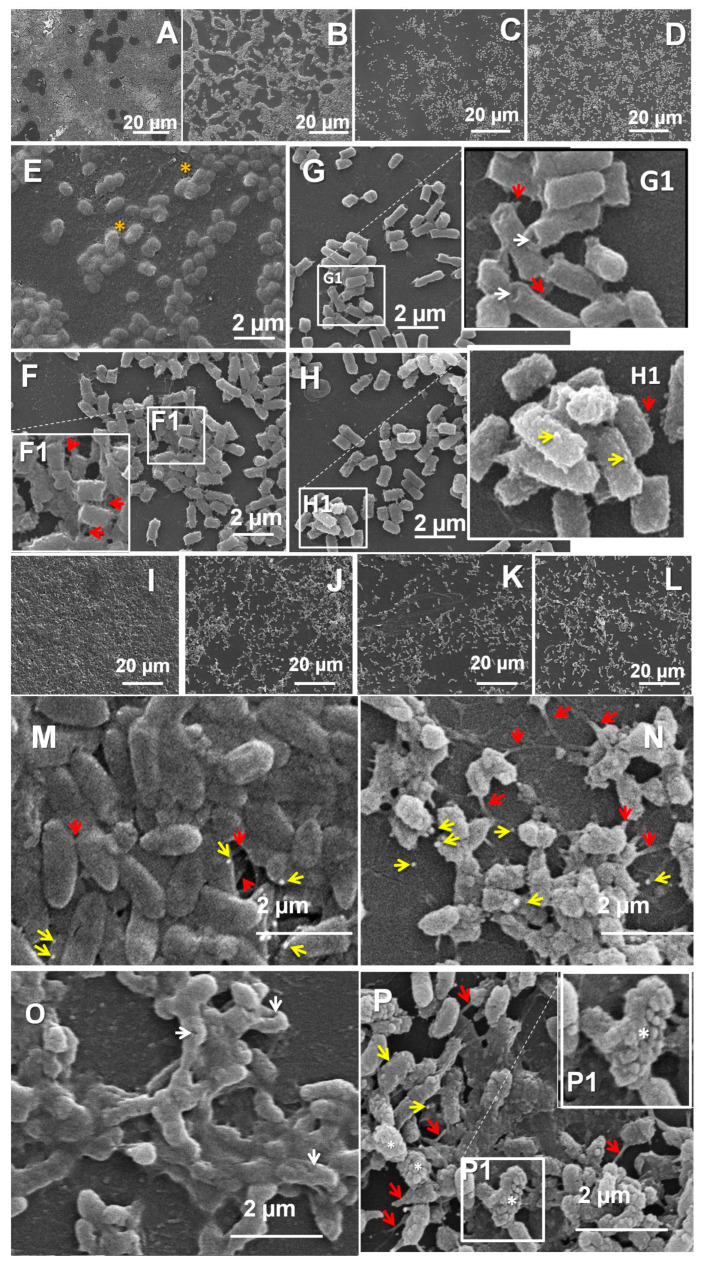
Scanning electron microscopy of biofilms of *E. coli* 25922 (**A**–**H**) and *P. aeruginosa* PAO1 (**I**–**P**). Arrows: white—infolds of the cell wall; yellow—outer membrane vesicles; red—tunneling nanotubules. (**A**) *E. coli* 48 h control biofilm; (**B**,**E**,**F**,**F1**) *E. coli* 24 h biofilm treated for a further 24 h with empty MPMs 3:1; yellow asterisk mark slimy covering of cells in some areas of the treated biofilm. (**G**,**G1**) *E. coli* 24 h biofilm treated for a further 24 h with CF-loaded MPMs 3:1; (**H**,**H1**) *E. coli* 24 h biofilm treated for a further 24 h with AZ-loaded MPMs 3:1. (**I**,**M**) *P. aeruginosa* 48 h control biofilm; (**J**,**N**) *P. aeruginosa* 24 h biofilm treated for a further 24 h with empty MPMs 1:1; (**K**,**O**) *P. aeruginosa* 24 h biofilm treated for a further 24 h with CF-loaded MPMs 1:1; (**L**,**P**,**P1**) *P. aeruginosa* 24 h biofilm treated for a further 24 h with AZ-loaded MPMs 1:1; white asterisks, cells with extensively blebbed surfaces.

To judge the success of the here-tested nanoparticles against the biofilms’ protective barriers of the two strains, it was important to analyze also the effects of the micelles on the biofilm bacterial cells. For this purpose, a SEM investigation was performed. The 24 h biofilms of the two model strains, *E. coli* 25922 and *P. aeruginosa* PAO1, were treated for a further 24 h with the pre-selected MPMs, respectively. 1:3 or 1:1, empty or antibiotic-loaded (Figure 9). Low-magnification images (Figure 9A–D,I–L) illustrate the significant reduction of the biofilm layers and the substratum coverage. Examination of the biofilms at higher magnifications showed differences in the cellular response between the two strains.

As determined by the CV and the viability tests, the MPMs that were most effective against *E. coli* biofilms were with a composition of 3:1. The SEM images taken after treatment with the empty micelles 3:1 showed a heterogeneity of the exfoliation effect. In some areas the cells were covered with a slimy layer of extracellular material (Figure 9E). The cell density in such areas was lower than that of untreated biofilms, but the cell morphology seemed unchanged. Other areas of this biofilm lacked a slimy cover. The cells in such loci were characterized by the occurrence of numerous protrusions (Figure 9F) similar to the ones described in the literature as “tunneling nanotubules” [51,52] which often connected closely situated bacteria. In the presence of the CF-loaded MPMs 3:1, such nanotubules were sparse, short, and in most cases did not connect neighboring cells. As a general aberration, deep invaginations occurred at the cell poles (Figure 9G,G1). In the presence of AZ, the treated cells again produced fewer nanotubules than the ones in biofilms treated with empty MPMs 3:1. In these samples, the cell surface was extensively blebbed (Figure 9H,H1).

The data from the CV and the Alamar blue tests indicated that the MPMs of choice for *P. aeruginosa* were these with the composition 1:1. The SEM results showed that, as a peculiarity of the untreated *P. aeruginosa* biofilms, the cells produced extracellular membrane vesicles (EVs) visible in both the untreated (Figure 9M) and the treated biofilms. Tubular interconnections between neighboring cells were also present in untreated control samples, though they were short due to the high cell density of the biofilm. The empty MPMs lowered the cell density, and the bacteria developed longer, thin nanotubules and released more EVs (Figure 9N). In the biofilm treated with the CF-loaded MPMs 1:1, EVs and nanotubules were almost completely lacking. Unlike with *E. coli*, infolds on the surface of *P. aeruginosa* occurred mainly on the lateral cell aspects (Figure 9O). The bacteria treated with AZ-loaded MPMs 1:1 were characterized by the presence of some nanotubules. In response to this micellar composition, together with the release of some EVs, the biofilm cells of PAO1 produced numerous surface blebs, indicating serious alterations of the integrity of the cell envelope (Figure 9P,P1).

In spite of the different extent of the alterations observed between the two bacterial species, there could be differentiated two distinct morphological patterns related to the two antibiotics loaded onto the micelles. These could be related to the specific mode of action of the two drugs but also to their different interactions with the polymeric carrier. Due to its zwitterionic nature, CF interacts electrostatically with the polycationic shell of MPMs. This in turn results in very high EE and sustained release of the drug (Figure 2 and Figure 4). CF, however, neutralizes a part of the cationic polymer shell (more precisely the PDMAEMA segments) and respectively decreases the ζ potential of the loaded MPMs. Thus, the PEO macromolecules have an important role in enhancing the penetration of the CF-loaded MPMs thought the bacterial wall, as previously has been shown [26]. Once entered and released externally, the fluoroquinolone CF exerts its bacteriostatic activity by inhibiting the enzyme DNA gyrase, which results in DNA supercoiling and hence blocking of the processes of transcription and replication [53,54].

The macrolide AZ inhibits protein synthesis at the level of translation. This is achieved by a reversible cutting of the 50S bacterial ribosomal subunit [55,56]. AZ interacts with the bacterial outer membrane and, by displacement of divalent cations from their binding sites on the LPS, interferes with the cell permeability [57,58]. This drug has also been shown to interfere with the bacterial virulence by modulating the expression of quorum sensing-regulated virulence factors [59]. Its “unconventional” characteristics as an antibacterial include also anti-inflammatory properties [56]. As shown above (Section 3.2), AZ is loaded in the micellar core by hydrophobic interactions. Due to its low miscibility with PPO (see Table 1) it is predominantly located in the PCL domains of the micellar core, resulting in a burst release of the drug, especially at MPMs 1:1 and 1:3. (see Figure 4). This might seem in apparent contradiction with the registered progress of the exfoliation effect from hour 4 to hour 24 for these compositions (Figure 6). The AZ-loaded MPMs are characterized by higher ζ potential values compared with those loaded with CF. Therefore, it can be suggested that the driving process of interaction at these systems is a strong electrostatic interaction with the negatively charged bacterial wall. Therefore, it can be hypothesized that once entered, the MPMs can interact with the polyanionic moieties in the biofilm matrix. Such are the alginate in *P. aeruginosa* biofilms and the colanic acid in the ECM of *E. coli*. Then they quickly release the AZ from the polymeric carrier inside the biofilm-forming ECM depot. In the course of the treatment, the amount of the biofilm and hence also the ECM are reduced, and this could lead to the gradual liberation of AZ from its ECM depot, thus prolonging the drug action with time.

One visual distinction between the action of the micelles loaded with the two antibiotics was the different type of alteration of the bacterial cell surfaces. In the presence of CF both strains had comparatively smooth areas on their surfaces interfered with by massive infolds, either polarly (*E. coli*) or on the side walls (*P. aeruginosa*) (Figure 9G,O). In the presence of AZ, the cell envelopes seemed to be quite activated by forming EVs and nanotubules (Figure 9H,P).

Nanotubules and extracellular vesicles (EVs) akin to those observed during the present SEM investigation are already well-recognized as structures related to bacterial communications at the cellular level. Unlike the regulatory mechanisms of the quorum sensing communication, however, the EVs and nanotubules have a different purpose—the possibility for contact-mediated transport allowing exchange of molecules and cytoplasmic components from cell to cell [52,60,61]. EVs are formed by budding of the outer, or the inner and outer membranes of the bacterial cell envelope and may carry materials with periplasmic or cytoplasmic origin, including metabolites, signaling molecules, virulence factors, etc. [61]. The released EVs can fuse with other cells and deliver their cargo. While enclosed in the EVs, this cargo is protected from the action of potentially harmful factors in the micro-environment [52]. Nanotubules emerge from conserved components of the flagellar export apparatus [62]. While facilitating intercellular interactions via their transporting capability, they can help the bacterial cells to overcome nutrient deprivation and other types of environmental stresses [51,63]. The observed occurrence of EVs and nanotubules here implies an attempt by the biofilm bacteria to at least partially compensate for the action of AZ and hence could be accepted as an adaptive strive.

### 3.5. Effects of MPMs on an Ex Vivo Skin Model of P. aeruginosa PAO1 Biofilm Infection

Due to the cationic nature of the micellar shell, the physicochemical parameters of the tested MPMs are sensitive to changes in pH or ionic strength [64]. Hence they are mainly applicable to lesions situated at the body surface or for decontamination of surfaces in hospitals [22,23]. Both pathogens that were addressed in this study have been related to skin infections such as diabetic foot or burn wounds [2]. Skin infections are predominantly accompanied by the formation of biofilms [2]. Taking into account their clinical relevance on the one hand and the need to minimize the use of laboratory animals in the experiments on the other, in vitro and ex vivo wound-infection models have lately been suggested in several publications (e.g., [35,65]).

**Figure 10:**
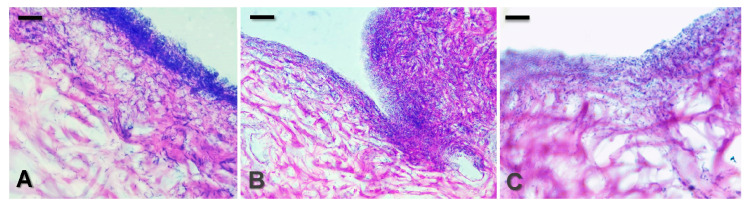
Histological sections of skin explants infected with *P. aeruginosa* PAO1 biofilm. (**A**) Untreated 24 h ex vivo biofilm. (**B**,**C**) Mature 24 h biofilms on skin explants were treated for 24 h with 0.25 mg mL^−1^ of MPMs 1:1 loaded with CF (**B**) or AZ (**C**). Bar = 10 µm.

In this study, an ex vivo model of *P. aeruginosa* skin infection was developed. The validity of the model was confirmed by the positive cultures of *P. aeruginosa* on its selective medium, Cetrimide agar. Histology showed the presence of extensive 24 h biofilm on the control skin samples (Figure 10A), similarly to the study of Seo et al. [66] exploiting the biofilm formation on porcine skin explants. Although the biofilm was clearly outlined, we observed that the bacteria could penetrate deeper in the skin explants. Previous studies have demonstrated that *P. aeruginosa* biofilms indeed can invade the dermis; however, the structure of biofilms formed in vivo and on ex vivo tissue varied from those grown in vitro [66,67] due to the appearance of the tunnel-like structures. The possibility of influencing this pre-formed skin biofilm infection by the application of the antibiotic-loaded micelles 1:1 was investigated. Figure 10 illustrates the good potential of the MPMs by showing the significant biofilm reduction after 4 h of treatment with CF-loaded (Figure 10B) or AZ-loaded (Figure 10C) MPMs 1:1.

### 3.6. MPM 1:1 Loaded with CF Sustained NO Production in Ex Vivo Murine Skin Explant P. aeruginosa Biofilm Model

NO is generated by activated non-immune cells, including keratinocytes, epithelial and endothelial cells, and innate immune cells (macrophages, monocytes, mast cells, and granulocytes), and is a part of the early pro-inflammatory response. It is a signaling molecule affecting the permeability of the skin barrier, wound healing processes, and antimicrobial defense. The pro-inflammatory effects of NO contribute to cutaneous inflammation via induction of the cyclooxygenase/prostaglandin pathway, migration of inflammatory cells, and local cytokine production [68]. However, in particular conditions, NO can have an opposite action and can inhibit cutaneous inflammation through inhibition of T cell proliferation and leukocyte migration/infiltration, enhancement of T cell apoptosis, as well as through down-regulation of cytokine production [68,69]. Skin manifestations are common, either representing local inoculation or secondary skin seeding following bloodstream infections [70], and occur primarily in necrotizing skin and soft tissue infections in diabetic, alcoholic, and immunocompromised patients.

**Figure 11:**
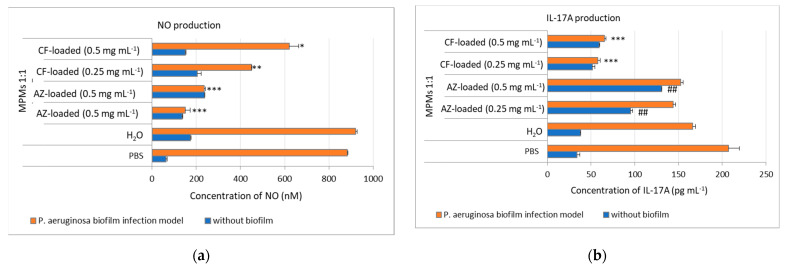
Effect of MPMs loaded with CF or AZ on NO (**a**) and IL-17A (**b**) production in ex vivo murine skin explant *P. aeruginosa* PAO1 biofilm model. Murine skin explants were infected with *P. aeruginosa* for 24 h for the development of biofilm. Afterwards the skin explants were treated with 50 µL of either 0.5 or 0.25 mg mL^−1^ MPMs loaded with CF or AZ. Control samples, infected or uninfected with *P. aeruginosa* biofilm, were treated in parallel with either PBS or dH_2_O (the solvent for the MPM samples). Data represents mean ± SD from 3 samples/group * *p* < 0.05, ** *p* < 0.01, *** *p* < 0.001 when comparing the biofilm groups to the control PBS one, ANOVA test; ## *p* < 0.05 when comparing the non-biofilm groups to the control PBS one, ANOVA test.

In our study we evaluated NO production on ex vivo murine skin explants in the *P. aeruginosa* biofilm model. We used ex vivo murine skin explants to induce biofilm formation with *P. aeruginosa* for 24 h. The explants were then treated within the next 24 h with MPMs 1:1 loaded with CF or AZ at concentrations of 0.5 and 0.25 mg mL^−1^. NO production in the explant’s culture medium was evaluated by Griess assay after treatment (Figure 11a).

We observed that NO was significantly elevated in cultures of ex vivo murine explants with *P. aeruginosa* biofilms in both PBS- and H_2_O-treated control samples (Figure 11a). We proposed that the biofilm formation might be a source of bacterial antigen, which could promote inflammation and consequently can explain the increased secretion of NO in the cultures. MPMs 1:1 loaded with both antibiotics decreased significantly the NO secretion in a manner dependent on the antibiotics. This inhibitory effect on NO was stronger when AZ was used in the nanocomposites but not CF (Figure 11a). Encapsulated CF can sustain NO in the biofilm explants, resulting probably in mounting inflammation, including cytokine production and adjusting the anti-infection innate response in keratinocytes. Indeed, a study showing the therapeutic effects of NO donors in animal models has demonstrated that NO can reduce microbial infection [71]. AZ did not show such an effect and markedly reduced NO production by the biofilm explants (Figure 11a). Low NO level was observed when topical application of cinnamaldehyde was used to inhibit *P. aeruginosa*-infected skin wounds [72], indicating that low NO might be important not only for inhibiting *P. aeruginosa* infection but also for healing of the wounds.

### 3.7. MPMs 1:1 Loaded with CF Inhibited IL-17A Production in Ex Vivo Murine Skin Explants P. aeruginosa Biofilm Model

Non-immune cells, in particular keratinocytes, as well as immune cells, can produce IL-17A during inflammation. Despite the fact that IL-17 has a key role in early anti-inflammatory response, its role in skin inflammation might be more complex, as the activation of IL-17 and IL-22 receptors on keratinocytes amplifies inflammatory responses in chronic skin diseases such as psoriasis [73], and exogenous IL-17A can compromise the proper skin wound healing process [74]. Recently it has been shown that cutaneous wound healing, along with wound inoculation with *Staphylococcus aureus* or *P. aeruginosa*, was exacerbated by infection-induced IL-17 and IL-22 production [75].

In our study we evaluated the secretion of IL-17A by ex vivo murine skin explants with *P. aeruginosa* biofilms and how the application of the MPMs 1:1 loaded with CF or AZ for 24 h affects that cytokine production (Figure 11b). We observed that biofilm formation with *P. aeruginosa* induced IL-17A secretion (PBS group, Figure 11b) that was unchanged after the application of MPM 1:1 (Figure 11b). When the MPMs 1:1 were loaded with antibiotics, the IL-17A level was elevated even in the absence of *P. aeruginosa* and became significantly higher in the AZ-loaded groups. In the latter group biofilm-induced IL-17A production was slightly decreased by the opposite of the nanocomposite loaded with CF (Figure 11b). However, the nanocomposite loaded with CF at the two concentrations (0.25 and 0.5 mg mL^−1^) showed similar inhibitory effects on IL-17A levels.

The data showed that MPMs loaded with CF or AZ had different effects on IL-17A production by the ex vivo skin explants. The MPMs loaded with AZ promoted IL-17 production even in the absence of the infection, suggesting that the release of AZ by the MPMs might have a direct effect on the skin explants. In contrast to healthy skin, AZ can decrease IL-17 production in psoriasis-like skin inflammation, proposing a distinct role of IL-17 in inflamed and normal skin [76]. In the biofilm samples, AZ failed to significantly inhibit the IL-17 levels, most likely because it stimulated the formation of EVs and nanotubules, which could trigger the cytokine production or IL-17-dependent signaling. However, the effect of MPM—AZ on IL-17 did not compromise its action on the biofilm destruction.

The MPMs loaded with CF inhibited the IL-17 levels, probably because they act directly on the biofilm-generated *P. aeruginosa* components, in turn, reducing the innate response to the infection (including IL-17 production). In this scenario, CF can reduce the ECM release. Indeed, CF may act on IL-17 in a more complex manner because the inhibition of IL-17 can ameliorate keratinocyte-borne cytokine responses in atopic dermatitis [77]. Hence, we strongly speculated that our nanocomposite loaded with CF can have a beneficial effect not only on destructing *P. aeruginosa* biofilm but also on limiting the IL-17-dependent cutaneous inflammation. However, more investigations are needed to prove this hypothesis.

## 4. Conclusions

The results showed that the MPMs were capable of destructing the biofilms of the two Gram-negative bacteria, and the viability experiments supported drug delivery. The positive results obtained with the two laboratory model strains, *E. coli* 25922 and *P. aeruginosa* PAO1, were also confirmed on clinical isolates of the two species. This supported the hypothesis of the study that the examined mixed polymeric micelles have good prospects as drug-delivery systems. Due to their cationic nature, the MPMs are sensitive to interactions with the anionic species present in physiological media, which could influence their physicochemical parameters (size, surface charge, colloidal stability, etc.). The latter is a limitation for their wider-scale applicability; however, they have good potential for topical application on skin biofilm infections as well as for disinfection of surfaces in healthcare and food-processing facilities. The biofilm response to the micelles loaded with the two antibiotics revealed two distinct patterns of action. One was at the level of both bacterial cell-structural alterations (as shown by SEM), and the other was at the level of interaction with host tissues (as demonstrated using an ex vivo biofilm infection model on skin samples, with experiments for production of NO and IL-17A). In addition to this, variation in the species- and strain-specific effectiveness was noted between the different types of micelles. They could be related to the different types of ECM molecules of the two species and also in their proportions in a particular biofilm. This requires facilitating the development of novel systems for directed drug-delivery. In order to further improve the drug-delivery systems, there is a need for future studies focused on the structure and biology of the ECM.

## Figures and Tables

**Table 1:** Solubility (δ) and polymer-drug miscibility (χ) parameters calculated for the hydrophobic core-forming polymers (PCL and PPO) and antibiotics (CF and AZ).

Polymer/Drug	δ [(MPa)1/2] *	χ_CF_	χ_AZ_
PCL	20.37	1.82	0.03
PPO	17.09	5.34	2.20
CF	24.87	-	-
AZ	20.03	-	-

* δ was calculated using Hoy molar attraction constants [29].

## Data Availability

The original contributions presented in this study are included in the article/Supplementary Material. Further inquiries can be directed to the corresponding authors.

## Supplementary Materials

The following supporting information can be downloaded at: https://www.mdpi.com/article/10.3390/microorganisms12122670/s1, Figure S1. Synthesis of poly(2-(dimethylamino)ethyl methacrylate)-b-poly(ε-caprolactone)-b-poly(2-(dimethylamino)ethyl methacrylate) triblock copolymer using PCL-based bifunctional macro-CTA (CTA-PCL-CTA) via RAFT polymerization; Figure S2. ^1^H NMR spectrum in CDCl3 of PCL-diol (blue), PCL-based macro-RAFT agent (red) and PDMAEMA-b-PCL-b-PDMAEMA triblock copolymer (black); Table S1. Molecular mass characteristics of polymer precursors and triblock copolymer determined by SEC and ^1^H NMR; Figure S3. Variation of critical solution concentration with polymeric micellar composition; Figure S4. Variation of critical solution concentration with polymeric micellar composition; Figure S5. DLS correlation functions of SCPMs and MPMs prepared from PDMAEMA-PCL-PDMAEMA and Pluronic F127 triblock copolymers in the concentration range of 1 to 0.125 mg.mL^−1^; Figure S6. Size distribution curves of SCPMs and MPMs prepared from PDMAEMA-PCL-PDMAEMA and Pluronic F127 triblock copolymers at a molar ratio of 1:1 in the concentration range of 1 to 0.125 mg.mL^−1^; Table S2. Hydrodynamic diameter, D_h_, and ζ-potential of SCPMs and MPMs based on PDMAEMA-PCL-PDMAEMA and Pluronic F127 triblock copolymers determined in a concentration range of 1 to 0.125 mg mL^−1^; Table S3. Encapsulation efficiency for different micellar compositions and the corresponding concentration of CF and AZ. Figure S7. Drug release profiles from SCPMs formed from PDMAEMA-PCL-PDMAEMA and Pluronic F127 triblock copolymers determined by HPLC. The release was performed at 37 °C in phosphate buffer pH 7.4. Each data point represents the arithmetic mean ± SD of three separate experiments. Figure S8. Changes of biofilm biomass after treatment of *E. coli* 25922 developed biofilms with mixed polymeric micelles unloaded or loaded with CF or AZ. The results are shown as percentage of the initial biofilm amount before treatment. Methodology: 96-well plate cultivation, crystal violet staining; Figure S9. Changes of biofilm biomass after treatment of *P. aeruginosa* PAO1-developed biofilms with mixed polymeric micelles unloaded or loaded with CF or AZ. The results are shown as percentage of the initial biofilm amount before treatment. Methodology: 96-well plate cultivation, crystal violet staining; Figure S10. Changes of biofilm biomass after treatment of *P. aeruginosa* PAO1 developed biofilms with mixed polymeric micelles unloaded, or loaded with CF or AZ; Table S4. Statistical significances (*p* < 0.05) of the differences between the biomass of the *E. coli* 25922 biofilms after treatment for 24 h with unloaded vs. antibiotics-loaded micelles. Methodology: ANOVA. Only data of micellar concentrations with low cytotoxicity are included in the table; Table S5. Statistical significances (*p* < 0.05) of the differences between the biomass of the *P. aeruginosa* PAO1 biofilms after treatment for 24 h with unloaded vs. antibiotics-loaded micelles. Methodology: ANOVA. Only data of micellar concentrations with low cytotoxicity are included in the table.
