# Supplementary material for: Gram Negative Biofilms: Structural and Functional Responses to Destruction by Antibiotic-Loaded Mixed Polymeric Micelles
Source: Microorganisms. 2024 Dec 23;12(12):2670. doi: 10.3390/microorganisms12122670 (PMC11728461; doi:10.3390/microorganisms12122670)
Supplement: Supplementary file 1 [file microorganisms-12-02670-s001.zip › microorganisms-3369092-supplementary.pdf]

SUPPLEMENTARY INFORMATION

GRAM NEGATIVE BIOFILMS: STRUCTURAL AND FUNCTIONAL RESPONSES TO DESTRUCTION BY ANTIBIOTIC-LOADED MIXED POLYMERIC MICELLES

Tsvetozara Damyanova[1*], Rumena Stancheva[2*], Milena Leseva[3], Petya A. Dimitrova[3], Tsvetelina Paunova-Krasteva[1], Dayana Borisova[1], Katya Kamenova[2], Petar Petrov[2], Ralitsa Veleva[3], Ivelina Zhivkova[5], Tanya Topouzova-Hristova[4]** Emi Haladjova[2]**, Stoyanka Stoitsova[1]**

* Authors with equal contribution

** Corresponding authors

1 Department of Microbiology, Stephan Angeloff Institute of Microbiology, Bulgarian Academy of Sciences, Akad. G. Bonchev st. 26, 1113 Sofia, Bulgaria
2 Institute of Polymers, Bulgarian Academy of Sciences, Akad. G. Bonchev st., bl. 103-A,,1113 Sofia, Bulgaria
3 Department of Immunology, Stephan Angeloff Institute of Microbiology, Bulgarian Academy of Sciences, Akad. G. Bonchev st. 26, 1113 Sofia, Bulgaria
4 Faculty of Biology, Sofia University "St. Kliment Ohridski", 8 Dragan Tsankov Blvd., 1164 Sofia, Bulgaria
5 National Reference Laboratory "Control and Monitoring of Antimicrobial Resistance" (NRL „CMAR"), Department of Clinical Microbiology, National Center of Infectious and Parasitic Disease, Yanko Sakuzov Blvd 26, 1504 Sofia, Bulgaria

1. *Synthesis of PDMAEMA-PCL-PDMAEMA triblock copolymer*

The amphiphilic PDMAEMA-b-PCL-b-PDMAEMA triblock copolymer was synthesized by two step RAFT procedure. In the first step a bifunctional CTA–PCL–CTA macro-RAFT agent was prepared. For this 2.5 g (0.31 mmol) and 0.21 g (0.93 mmol) CTA PCL-diol were placed in 100 ml one-neck round-bottom flask equipped with a magnetic stirring bar and dissolved in 35 mL freshly distilled DCM under inert atmosphere. After homogenization the solution was cooled down to 0°C using ice bath followed by addition of 0.19 g (0.03 mmol) DCC and 0.056 g (0.46 mmol) DMAP. The mixture was stirred for 20 minutes in ice-water bath, then the reaction temperature was raised slowly to 22 °C. The reaction was carried out under stirring for 48 hours at room temperature. Thereafter, the reaction mixture was filtered, the filtrate was concentrated and precipitated into a 10-fold excess of cold methanol (-30 °C). The final product (yield 85%) was isolated by filtering and drying under vacuum at 40 °C for 48 hours. In the second step 0.5 g (0.058 mmol) CTA–PCL–CTA and 1.9 mg (0.011 mmol) AIBN were dissolved in 8 mL anisole under inert atmosphere in 50 ml round-bottom flask equipped with a silicon septum. 0.82 mL DMAEMA (5.2 mmol) was then added and the reaction mixture was left stirring at room temperature under argon flow for 15 minutes. The polymerization was conducted for 24 h at 75 °C. After the reaction termination THF was added to dissolve the formed copolymer. The product (yield 75%) was isolated by precipitation in 10-fold excess of n-hexane and dried under vacuum for 48 h.

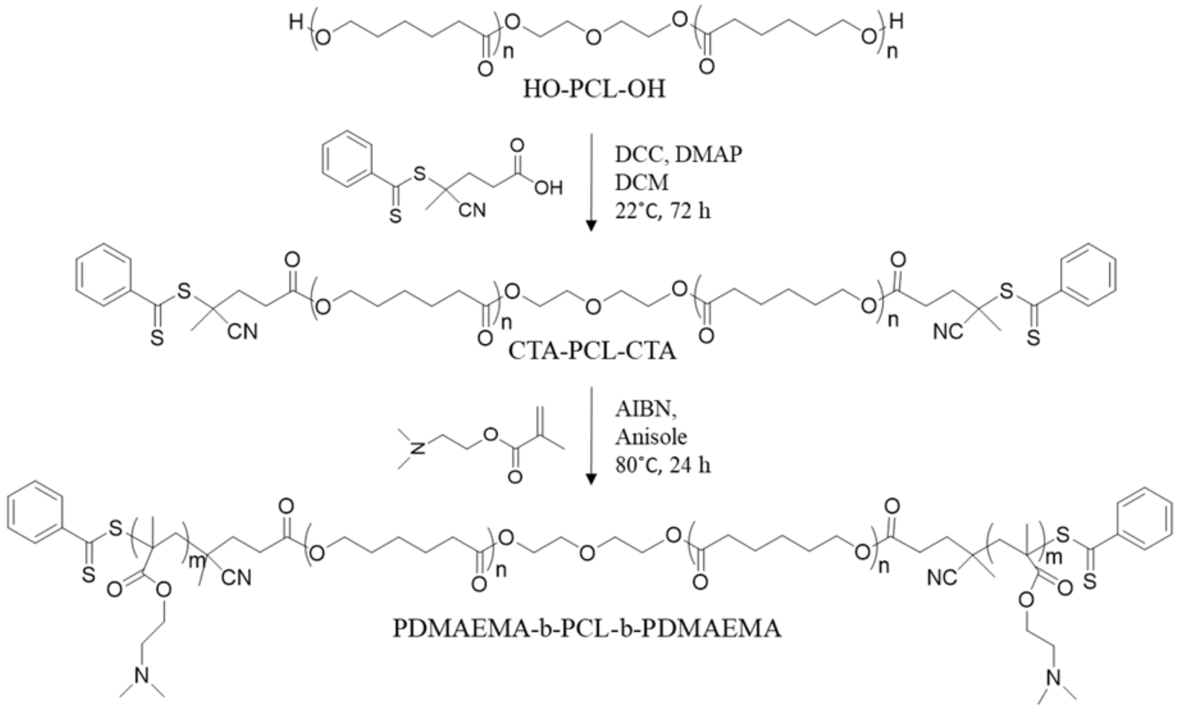

Figure S1. Synthetic route to poly(2-(dimethylamino)ethyl methacrylate)-b-poly(ε-caprolactone)-b-poly(2-(dimethylamino)ethyl methacrylate) triblock copolymer using PCL-based bifunctional macro-CTA (CTA-PCL-CTA) via RAFT polymerization.

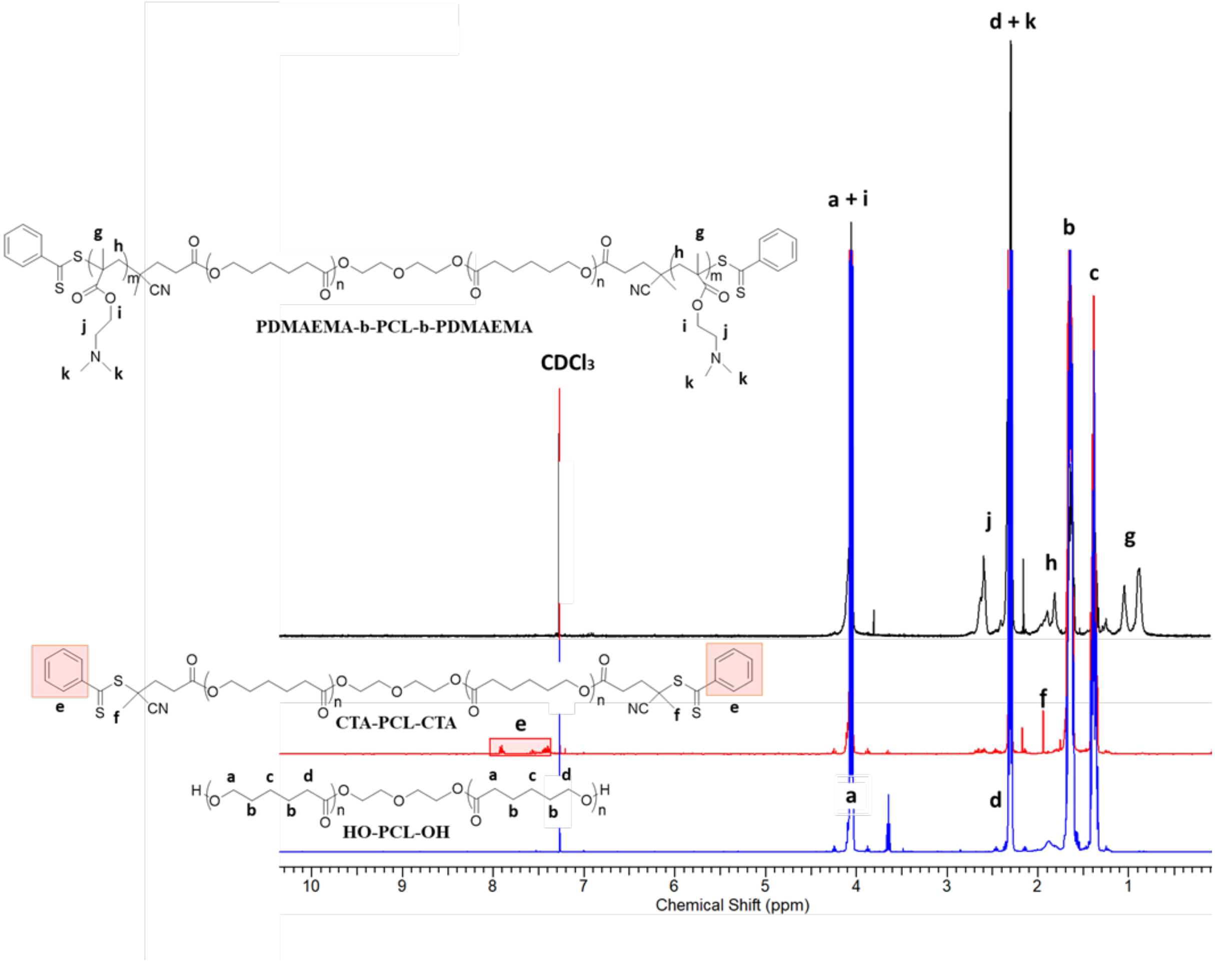

Figure S2. ^{1}H NMR spectrum in $CDCl_3$ of PCL-diol (blue), PCL-based macro-RAFT agent (red) and PDMAEMA-b-PCL-b-PDMAEMA triblock copolymer (black).

CTA-PCL-CTA macro-RAFT agent

^{1}H-NMR (CDCl3, δ ppm): e 7.9-7.4 (m, 10 H from phenyl groups), a 4.06–4.0 (m, 140 H, $-C\underline{H}_2-O-$), d 2.31 (m, 140 H, $-C(O)-C\underline{H}_2$ -), f 1.95 (m, 6H, $-C(N)-C-C\underline{H}_3$), b 1.65 (m, 280 H, $-C(O)-CH_2-C\underline{H}_2-CH_2-$), c 1.39–1.35 (m, 140 H, $-CH_2-C\underline{H}_2-CH_2-$).

PDMAEMA-b-PCL-b-PDMAEMA triblock copolymer

^{1}H-NMR (CDCl3, δ ppm): d (ppm) = a+i 4.06-4.00 (m, 140 H, $-C\underline{H}_2-O-$ from PCL+ m, 140 H, $–O–C\underline{H}_2–CH_2–N–$ from PDMAEMA), j 2.53 (s, 140 H, $-CH_2–C\underline{H}_2–N(CH_3)$), d,k 2.45-2.31(m, 140 H, $-C(O)-C\underline{H}_2$ - from PCL + m, 420 H, ($–N(C\underline{H}_3)$ from PDMAEMA), h 1.86–1.75 (140 H, $–C\underline{H}_2–C–CH_3$), b 1.65 (m, 280 H, $-C(O)-CH_2-C\underline{H}_2-CH_2-$), c 1.39–1.35 (m, 140 H, $-CH_2-C\underline{H}_2-CH_2-$), g 1.00–0.64 (m, 210 H, $–CH_2–C(CO)–C\underline{H}_3$).

Table S1. Molecular mass characteristics of polymer precursors and $PDMAEMA_{35}$-b-PCL_{70}-b-$PDMAEMA_{35}$ triblock copolymer determined by SEC and ^{1}H NMR.

**Sample**	**M_n g.mol^{-1}**		**Đ***
	^{1}H NMR	SEC	
**HO- PCL_{70}-OH**	8000	9700	1.96
**CTA- PCL_{70}-CTA**	8550	18500	2.00
**$PDMAEMA_{35}$-b-PCL_{70}-b-$PDMAEMA_{35}$**	18 900	20 308	1.81

*The value is determined from SEC.

2. Preparation and characterization of PMs

Determination of critical micellar concentration (CMC)

CMCs values were determined the dye solubilization method previously described in Refxx In brief, micellar dispersions of 2 mL at 10 different concentrations in the 0.001 - 0.5 mg.mL^{-1} range were prepared by diluting a stock solution. Then, 20 µL of 1,6-diphenyl-1,3,5-hexatriene (DPH) solution in methanol (0.4 mM) were added to each sample. The samples were incubated in the dark at room temperature. After 18 h UV–VIS absorption spectra of DPH were recorded in the λ=300–500 nm range. The CMC value of each sample was determined from the break of the absorbance intensity at 356 nm vs. concentration curve.

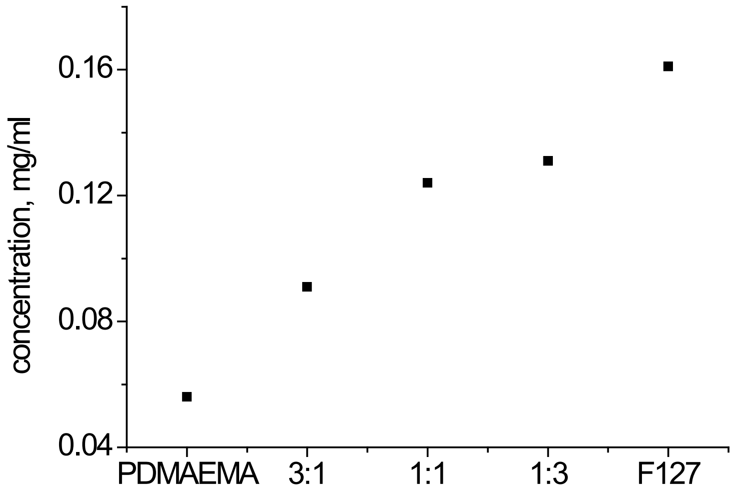

Figure S3. Variation of critical solution concentration with polymeric micellar composition…

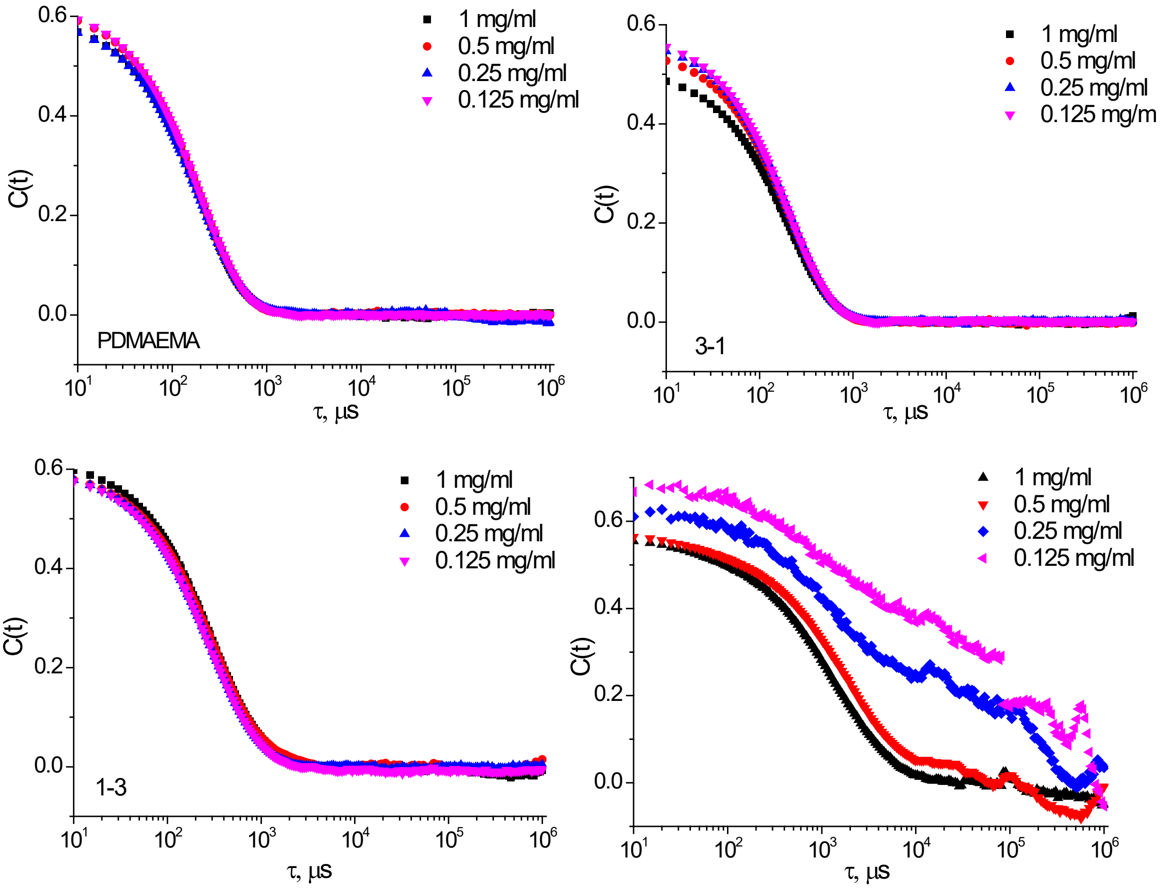

Figure S4. DLS correlation functions of SCPMs and MPMs prepared from PDMAEMA-PCL-PDMAEMA and Pluronic F127 triblock copolymers in the concentration range of 1 to 0.125 $mg.ml^{-1}$.

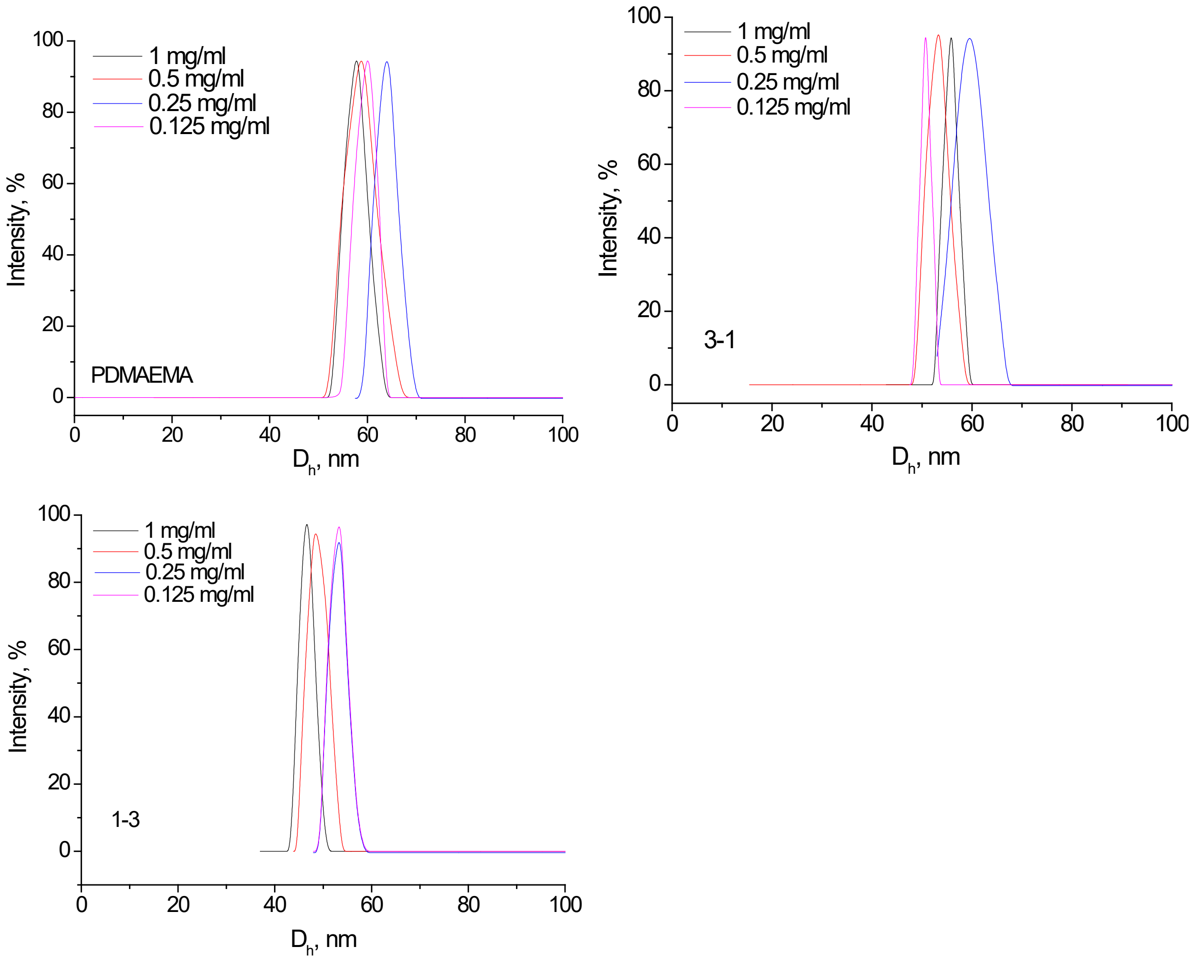

Figure S5. Size distribution curves of SCPMs and MPMs prepared from PDMAEMA-PCL-PDMAEMA and Pluronic F127 triblock copolymers at a molar ratio of 1:1 in the concentration range of 1 to 0.125 mg.ml^{-1}.

Table S2. Hydrodynamic diameter, D_h, and ζ-potential of SCPMs and MPMs based on PDMAEMA-PCL-PDMAEMA and Pluronic F127 triblock copolymers determined in a concentration range of 1 to 0.125 mg.ml^{-1}.

**Micellar composition**	**Concentration, mg/ml**							
	**1**	**0.5**	**0.25**	**0.125**	**1**	**0.5**	**0.25**	**0.125**
	D_h,nm				ζ-potential, mV			
**PDMAEMA**	35.6	35.0	33.5	37.7	31.3	34.9	33.3	32.4
**3-1**	39.5	39.8	42.5	36.7	30.0	31.1	31.2	28.9
**1-1**	34.4	32.7	34.1	37.6	25.0	24.3	25.1	24.6
**1-3**	36.9	36.2	37.0	36.3	20.4	21.1	23.2	22.5

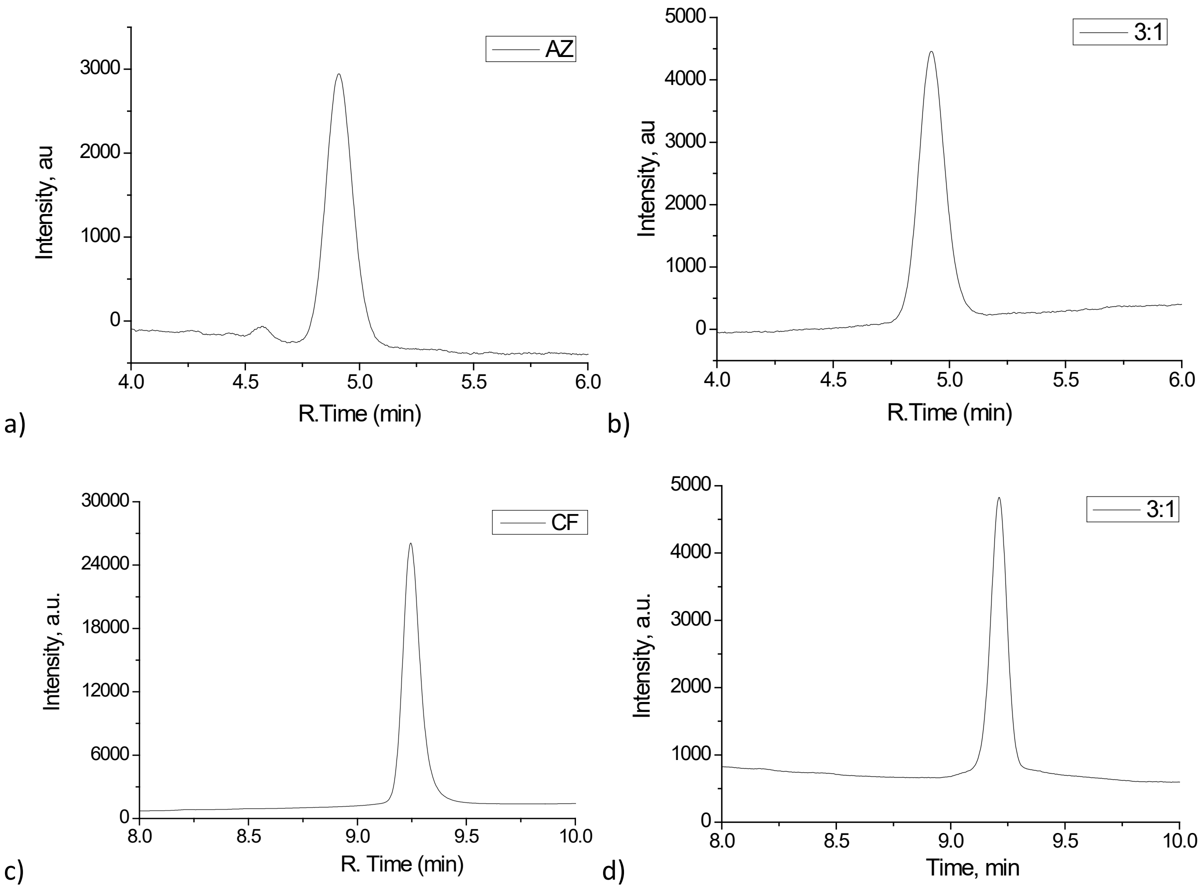

Figure S6. HPLC chromatograms of pure antibiotics used as standard (AZ (a) and CF (c)) and antibiotics extracted from MPMs composition 3:1 (AZ (b) and CF (d)). The samples were prepared at polymer to drug mass ratio 10:1. The integrated area of the peaks was used for calculation of EE and DLC.

Table S3. Encapsulation efficiency at polymer to drug mass ratio 10:1 for different micellar compositions and the corresponding concentration of CF and AZ.

Micellar composition	Encapsulation efficiency for CF, %	CF concentration, mg/ml	Encapsulation efficiency for AZ, %	AZ concentration, mg/ml
PDMAEMA	96,2	0,1058	73,3	0,0761
3-1	95,7	0,1005	51,6	0,0524
1-1	96,9	0,1042	41,8	0,0423
1-3	95,2	0,1023	33,8	0,0350

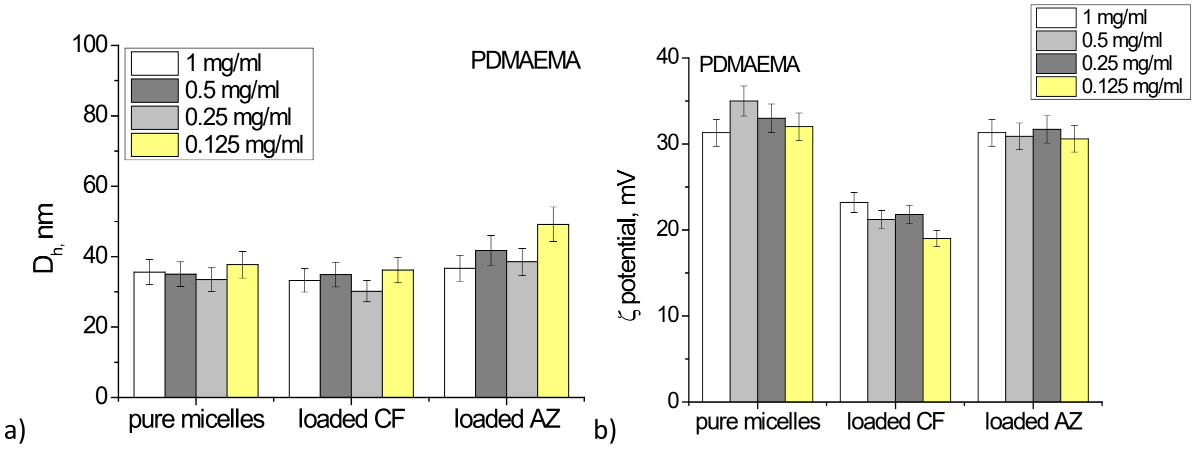

Figure S7. Size (a) and ζ potential (b) variations of SCPMs formed from PDMAEMA-PCL-PDMAEMA triblock copolymer. Each data point in represents the arithmetic mean ± SD of three separate experiments.

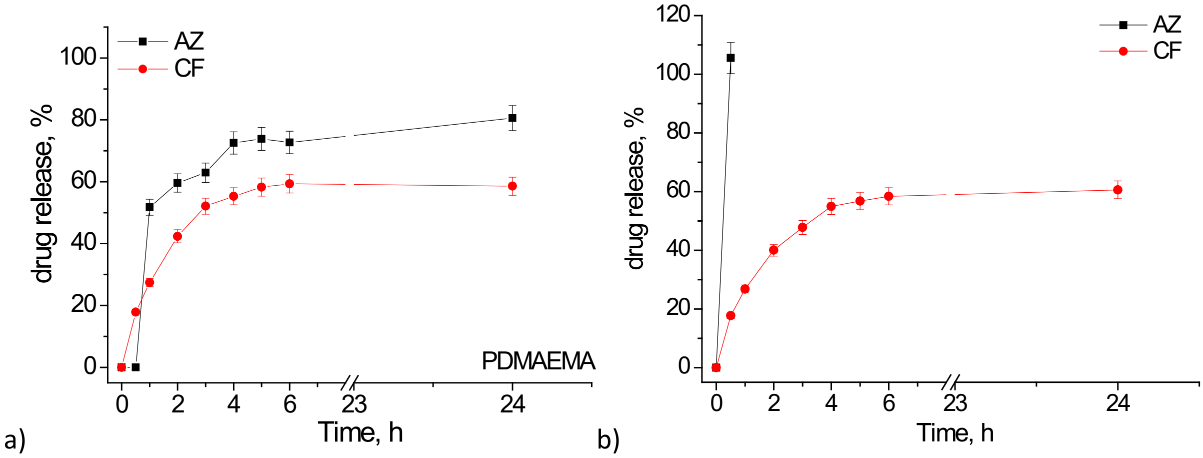

Figure S8. Drug release profiles of SCPMs formed from PDMAEMA-PCL-PDMAEMA (a) and Pluronic F127 (b) triblock copolymers determined by HPLC. The release was performed at 37 °C in phosphate buffer pH 7.4. Each data point in represents the arithmetic mean ± SD of three separate experiments.

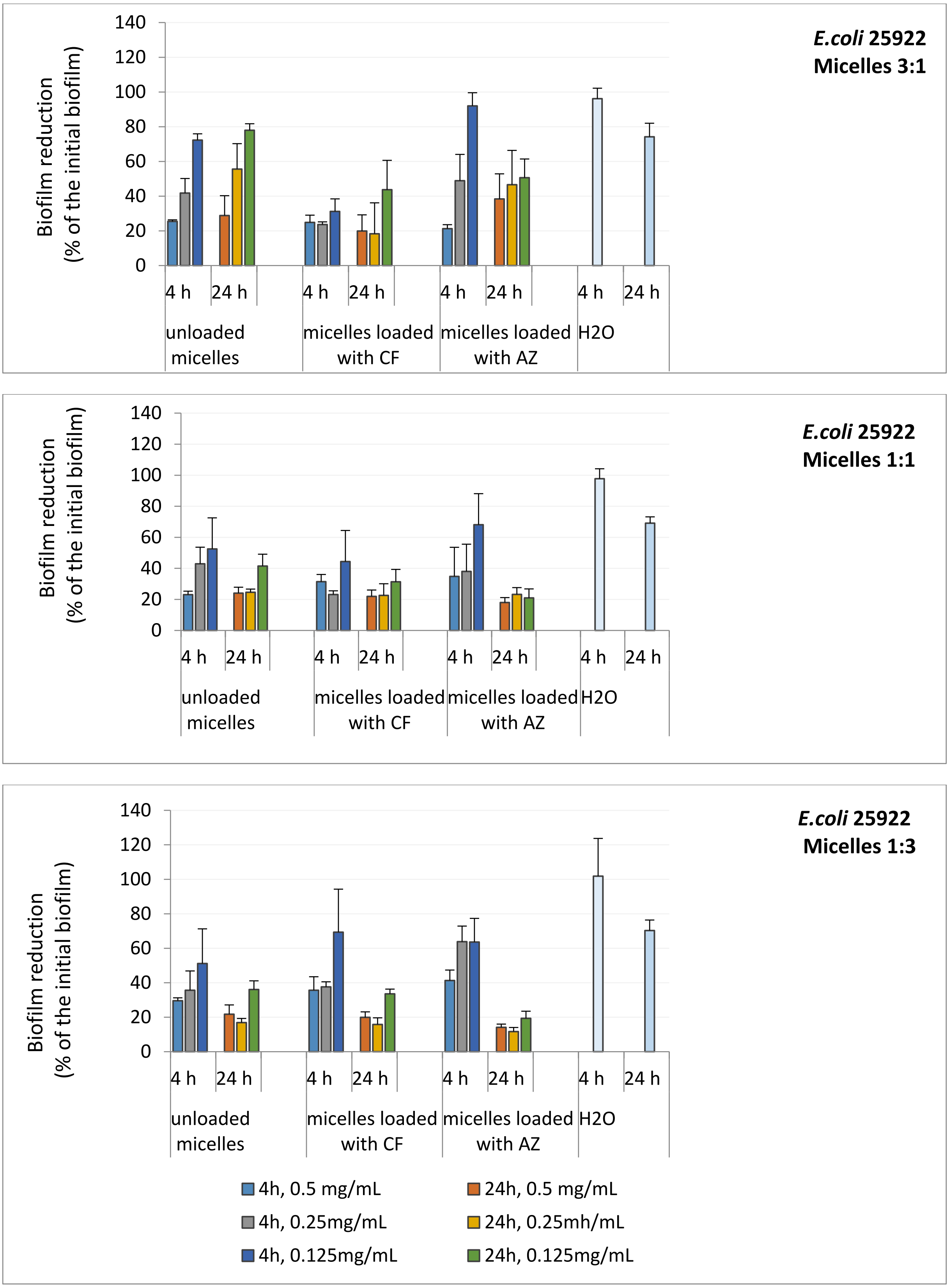

Figure S9 Changes of biofilm biomass after treatment of E. coli 25922 developed biofilms with mixed polymeric micelles unloaded, or loaded with CF or AZ. The results are shown as per

cent of the initial biofilm amount, before treatment. Methodology: 96-well plate cultivation, crystal violet staining.

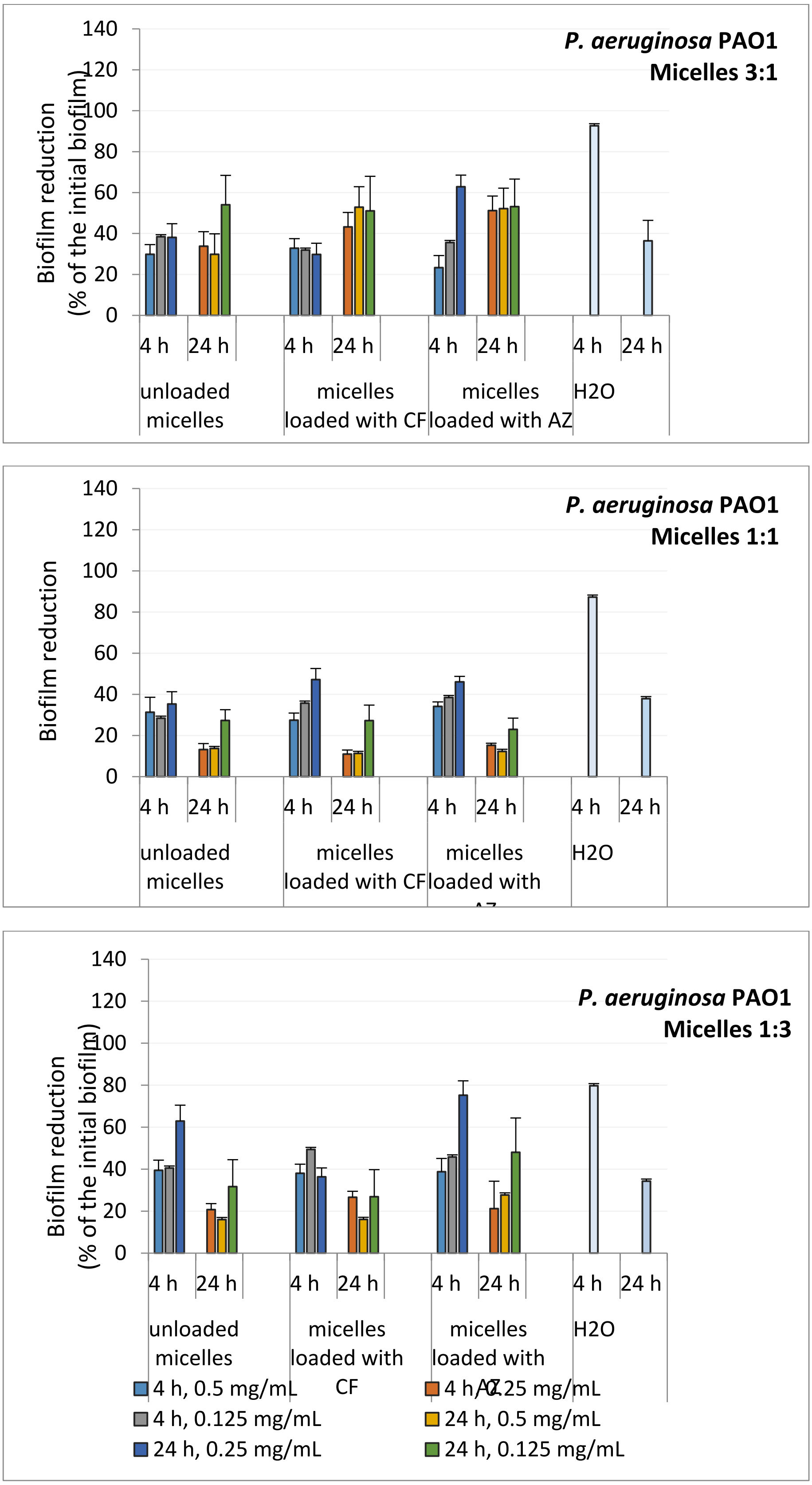

Figure S10. Changes of biofilm biomass after treatment of *P. aeruginosa* PAO1 developed biofilms with mixed polymeric micelles unloaded, or loaded with CF or AZ. The results are shown as per cent of the initial biofilm amount, before treatment. Methodology: 96-well plate cultivation, crystal violet staining.

Table S4. Statistical significances ($P \leq 0.05$) of the differences between the biomass of the *E. coli* 25922 biofilms after treatment for 24 h with unloaded vs. antibiotics-loaded micelles. Methodology: ANOVA. Only data of micellar concentrations with low cytotoxicity are included in the table.

<table>
<tr><td colspan="2" rowspan="2">Compared data sets: Horizontal: unloaded micelles, compared to vertical: antibiotic-loaded micelles</td><td rowspan="2"></td><td colspan="2">Unloaded micelles</td><td rowspan="2"></td><td colspan="2">Unloaded micelles</td><td rowspan="2"></td><td colspan="2">Unloaded micelles</td></tr>
<tr><td>0. 25 mg/mL</td><td>0.125 mg/mL</td><td>0. 25 mg/mL</td><td>0.125 mg/mL</td><td>0. 25 mg/mL</td><td>0.125 mg/mL</td></tr>
<tr><td rowspan="2">CF-loaded micelles</td><td>0.25 mg/ mL</td><td rowspan="4">Micelles 3:1</td><td>P<0.005 diminution</td><td></td><td rowspan="4">Micelles 1:1</td><td>NS</td><td></td><td rowspan="4">Micelles 1:3</td><td>NS</td><td></td></tr>
<tr><td>0.125 mg/ mL</td><td></td><td>P<0.001 diminution</td><td></td><td>NS</td><td></td><td>NS</td></tr>
<tr><td rowspan="2">AZ-loaded micelles</td><td>0.25 mg/ mL</td><td>NS</td><td></td><td>NS</td><td></td><td>P<0.05 diminuti on</td><td></td></tr>
<tr><td>0.125 mg/ mL</td><td></td><td>P<0.05 diminution</td><td></td><td>P<0.01 diminution</td><td></td><td>P<0.01 diminution</td></tr>
</table>

Table S5. Statistical significances ($P \leq 0.05$) of the differences between the biomass of the *P. aeruginosa* PAO1 biofilms after treatment for 24 h with unloaded vs. antibiotics-loaded micelles. Methodology: ANOVA. Only data of micellar concentrations with low cytotoxicity are included in the table.

Compared data sets: Horizontal: unloaded micelles, compared to vertical: antibiotic-loaded micelles		**Micelles 3:1**	**Unloaded micelles**		**Micelles 1:1**	**Unloaded micelles**		**Micelles 1:3**	**Unloaded micelles**	
			**0. 25 mg/mL**	**0.125 mg/mL**		**0. 25 mg/mL**	**0.125 mg/mL**		**0. 25 mg/mL**	**0.125 mg/mL**
**Loaded with CF**	**0.25 mg/mL**		**P<0.001 increase**			**P<0.05 diminution**			**NS**	
	**0.125 mg/mL**			**NS**			**P<0.05 diminution**			**NS**
**Натовар ени с AZ**	**0.25 mg/mL**		**P<0.001 increase**			**NS**			**P<0.05 increase**	
	**0.125 mg/mL**			**NS**			**P<0.05 diminution**			**P<0.05 increase**
